# Supplementary material for: Global patterns and rates of habitat transitions across the eukaryotic tree of life
Source: Nat Ecol Evol. 2022 Aug 4;6(10):1458–70. doi: 10.1038/s41559-022-01838-4 (PMC9525238; doi:10.1038/s41559-022-01838-4)
Supplement: Supplementary file 1 — Supplementary Figs. 1–16, Notes 1–3 and Tables 1–4. [file 41559_2022_1838_MOESM1_ESM.pdf]

nature portfolio

https://doi.org/10.1038/s41559-022-01838-4

Supplementary information

Global patterns and rates of habitat transitions across the eukaryotic tree of life

In the format provided by the authors and unedited

Global patterns and rates of habitat transitions across the eukaryotic tree of life

Supplementary Information

Mahwash Jamy[1], Charlie Biwer[1], Daniel Vaulot[2,3], Aleix Obiol[4], Hongmei Jing[5], Sari Peura[6,7], Ramon Massana[4], Fabien Burki[1,7*]

[1] Department of Organismal Biology (Systematic Biology), Uppsala University, Uppsala, Sweden

[2] Sorbonne Université, CNRS, UMR7144, Team ECOMAP, Station Biologique, Roscoff, France

[3] Asian School of the Environment, Nanyang Technological University, Singapore

[4] Department of Marine Biology and Oceanography, Institut de Ciències del Mar (ICM-CSIC), Barcelona, Spain

[5] CAS Key Lab for Experimental Study Under Deep-sea Extreme Conditions, Institute of Deep-sea Science and Engineering, Chinese Academy of Sciences, Sanya, China

[6] Department of Ecology and Genetics (Limnology), Uppsala University, Uppsala, Sweden

[7] Science for Life Laboratory, Uppsala University, Sweden

*Corresponding author: fabien.burki@ebc.uu.se

This pdf file contains

Supplementary Figure 1-16

Supplementary Notes 1-3

Supplementary Tables 1-4

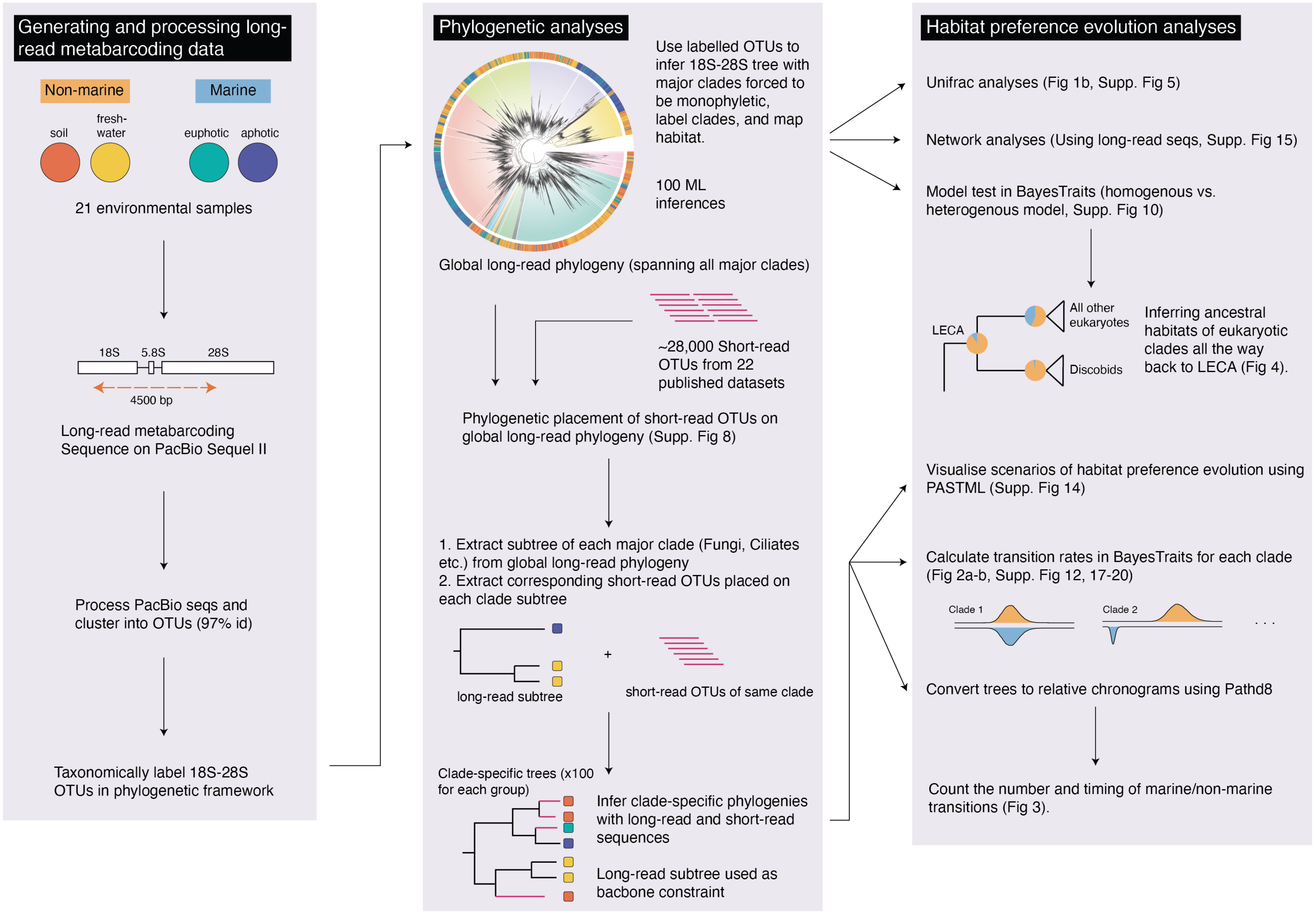
Generating and processing long-read metabarcoding data
Non-marine
Marine
soil
fresh-water
euphotic
aphotic
21 environmental samples
18S
5.8S
28S
4500 bp
Long-read metabarcoding Sequence on PacBio Sequel II
Process PacBio seqs and cluster into OTUs (97% id)
Taxonomically label 18S-28S OTUs in phylogenetic framework
Phylogenetic analyses
Use labelled OTUs to infer 18S-28S tree with major clades forced to be monophyletic, label clades, and map habitat.
100 ML inferences
Global long-read phylogeny (spanning all major clades)
~28,000 Short-read OTUs from 22 published datasets
Phylogenetic placement of short-read OTUs on global long-read phylogeny (Supp. Fig 8)
1. Extract subtree of each major clade (Fungi, Ciliates etc.) from global long-read phylogeny
2. Extract corresponding short-read OTUs placed on each clade subtree
+
long-read subtree
short-read OTUs of same clade
Clade-specific trees (x100 for each group)
Infer clade-specific phylogenies with long-read and short-read sequences
Long-read subtree used as bacbone constraint
Habitat preference evolution analyses
Unifrac analyses (Fig 1b, Supp. Fig 5)
Network analyses (Using long-read seqs, Supp. Fig 15)
Model test in BayesTraits (homogenous vs. heterogenous model, Supp. Fig 10)
LECA
All other eukaryotes
Discobids
Inferring ancestral habitats of eukaryotic clades all the way back to LECA (Fig 4).
Visualise scenarios of habitat preference evolution using PASTML (Supp. Fig 14)
Calculate transition rates in BayesTraits for each clade (Fig 2a-b, Supp. Fig 12, 17-20)
Clade 1
Clade 2
. . .
Convert trees to relative chronograms using Pathd8
Count the number and timing of marine/non-marine transitions (Fig 3).

Supplementary Figure 1. (On previous page). Broad overview of the analyses carried out in this study. Not all analyses (for example the comparison of long-read and short-read metabarcodes) are depicted.

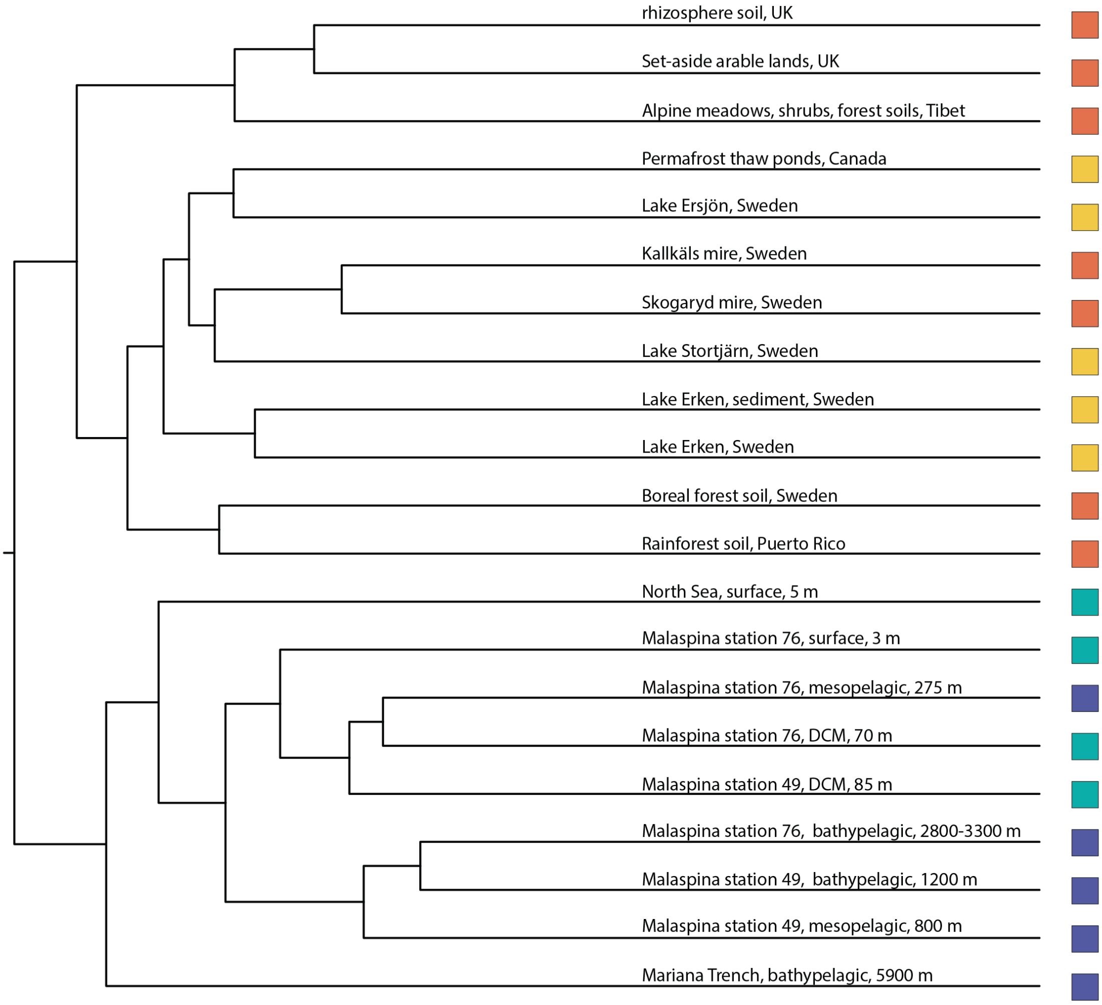

Supplementary Figure 2. Hierarchical clustering of the 21 samples sequenced with PacBio based on unweighted UniFrac analyses on the tree in Figure 1a. UniFrac measures the dissimilarity between communities by taking phylogenetic relatedness into account[1]. Here, soil=orange, yellow=freshwater, light blue=marine euphotic, and dark blue=marine aphotic samples. The figure shows a deep split between marine and non-marine communities. Furthermore, on average, marine communities seem to be more closely related to each other than non-marine communities.

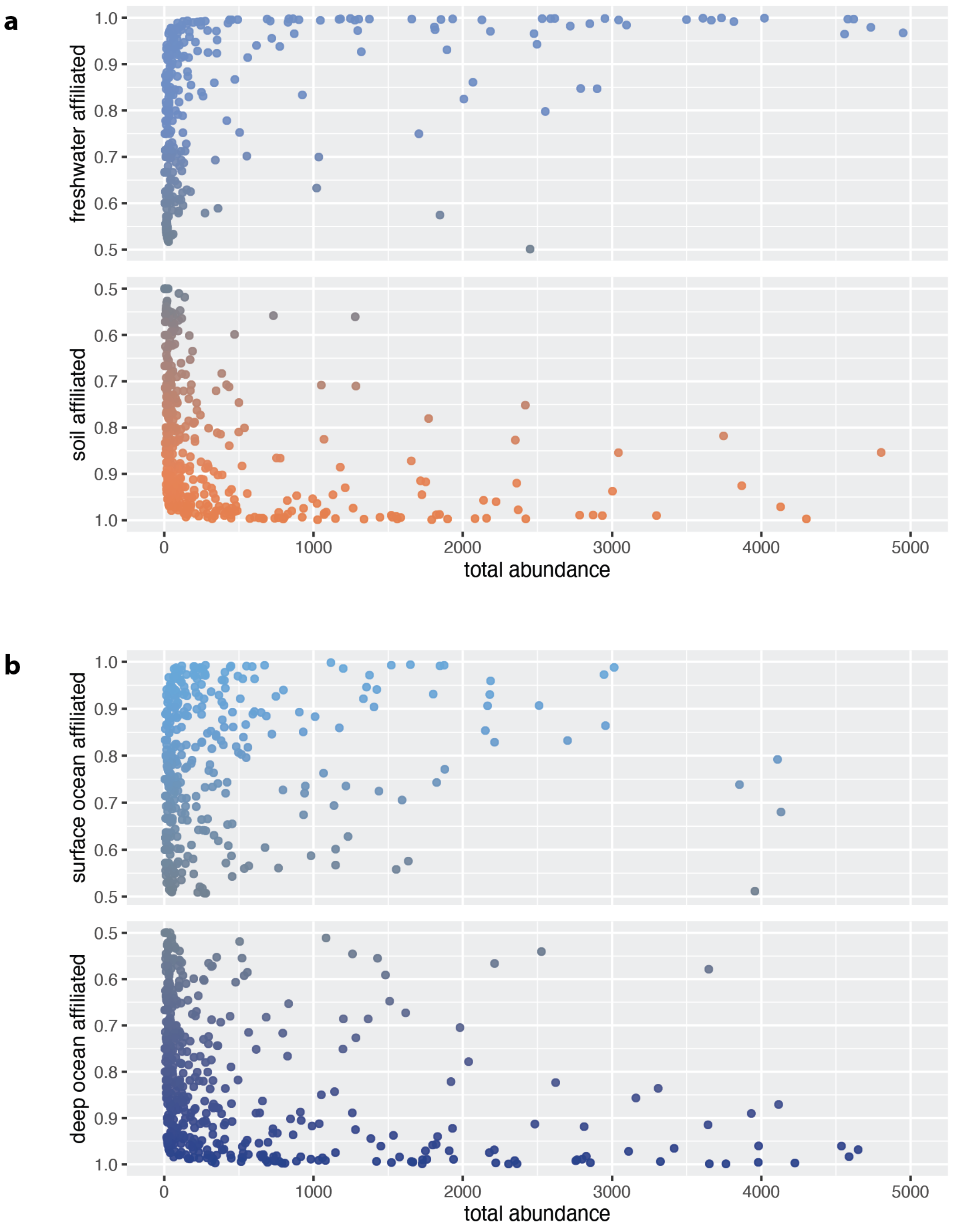

Supplementary Figure 3. Distribution patterns of **(a)** the 723 (out of 771) shared OTUs between freshwater and soil PacBio samples, and **(b)** the 801 (out of 854) shared OTUs between the surface and deep ocean. All OTUs were clustered at 97% similarity. The x-axis represents the total number of reads in the OTU (a cut-off of 5000 reads was chosen as shared OTUs with higher abundances were spurious), and the y-axis represents affiliation to each environment as measured by the (number of reads in a habitat) / (total number of reads). This analysis follows the reasoning presented in [2]. Briefly, shared OTUs do not necessarily represent generalists across two habitats, but can also represent contamination, i.e. taxa that ended up in another habitat due to run-off, water mixing etc. To differentiate between the two, the affiliation to each habitat can be measured. For instance, if a shared

OTU between soil and freshwater habitats has roughly half of its reads present in soil and half in freshwater, then it can be assumed that the taxon is more likely to be a generalist. However, if the shared OTU has 90% of its reads in soil habitats, and only 10% in freshwater, it is more likely that the taxon lives in soil, but ended up in freshwater due to run-off or some other way. The analysis shows a number of generalists in both **(a)** and particularly in **(b)**. As in Sieber et al. [2], "generalists" tend to be taxa with lower read abundances overall. Since the OTUs were clustered at 97% similarity, it is also possible that "generalists" are represented by closely related species with their own specific niches.

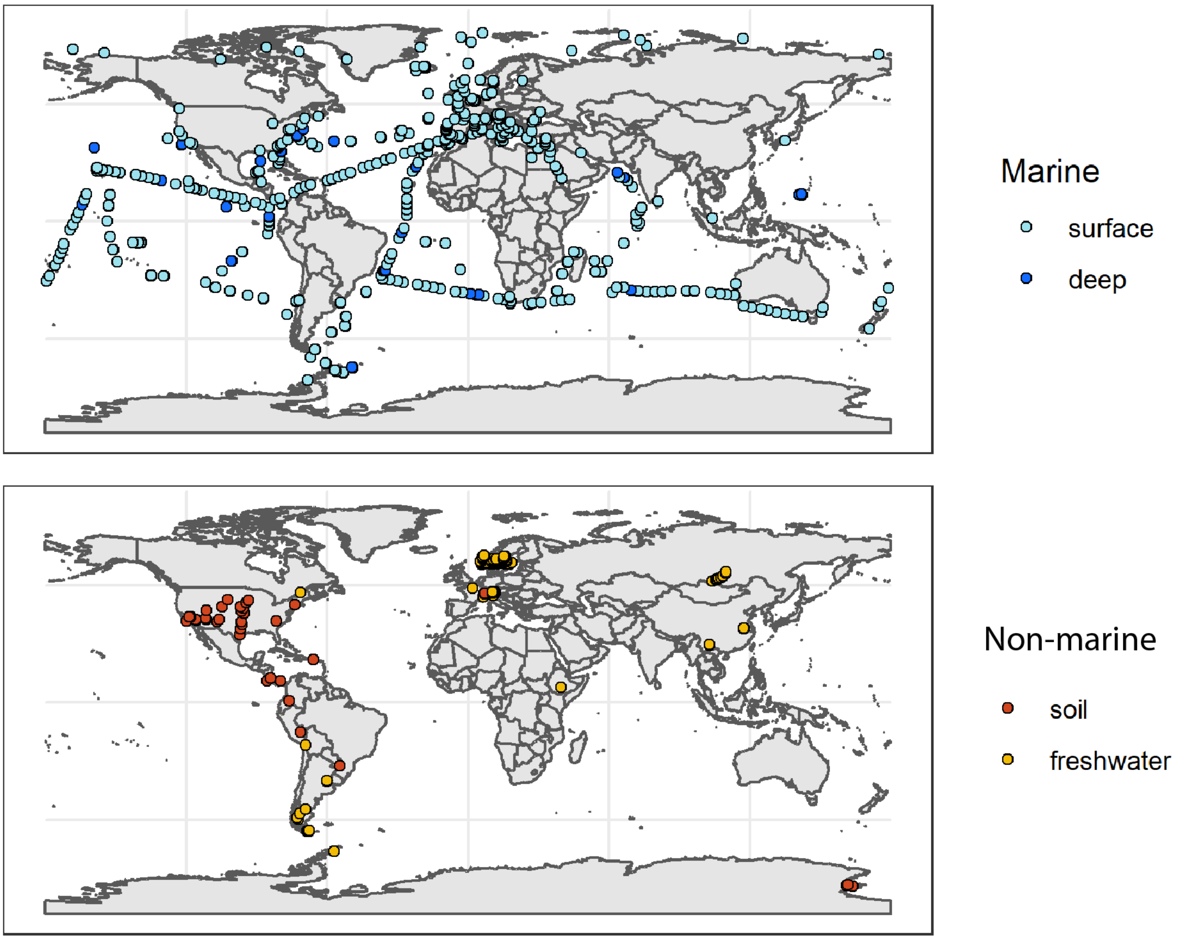

Supplementary Figure 4. Geographical locations corresponding to the short-read metabarcoding data. The upper panel showcases the provenance of marine samples, from the marine euphotic (light blue) and the marine aphotic (dark blue). The lower panel displays samples originating from non-marine habitats, with soil samples in red and samples from freshwater systems (lakes, ponds, creeks and rivers) in yellow.

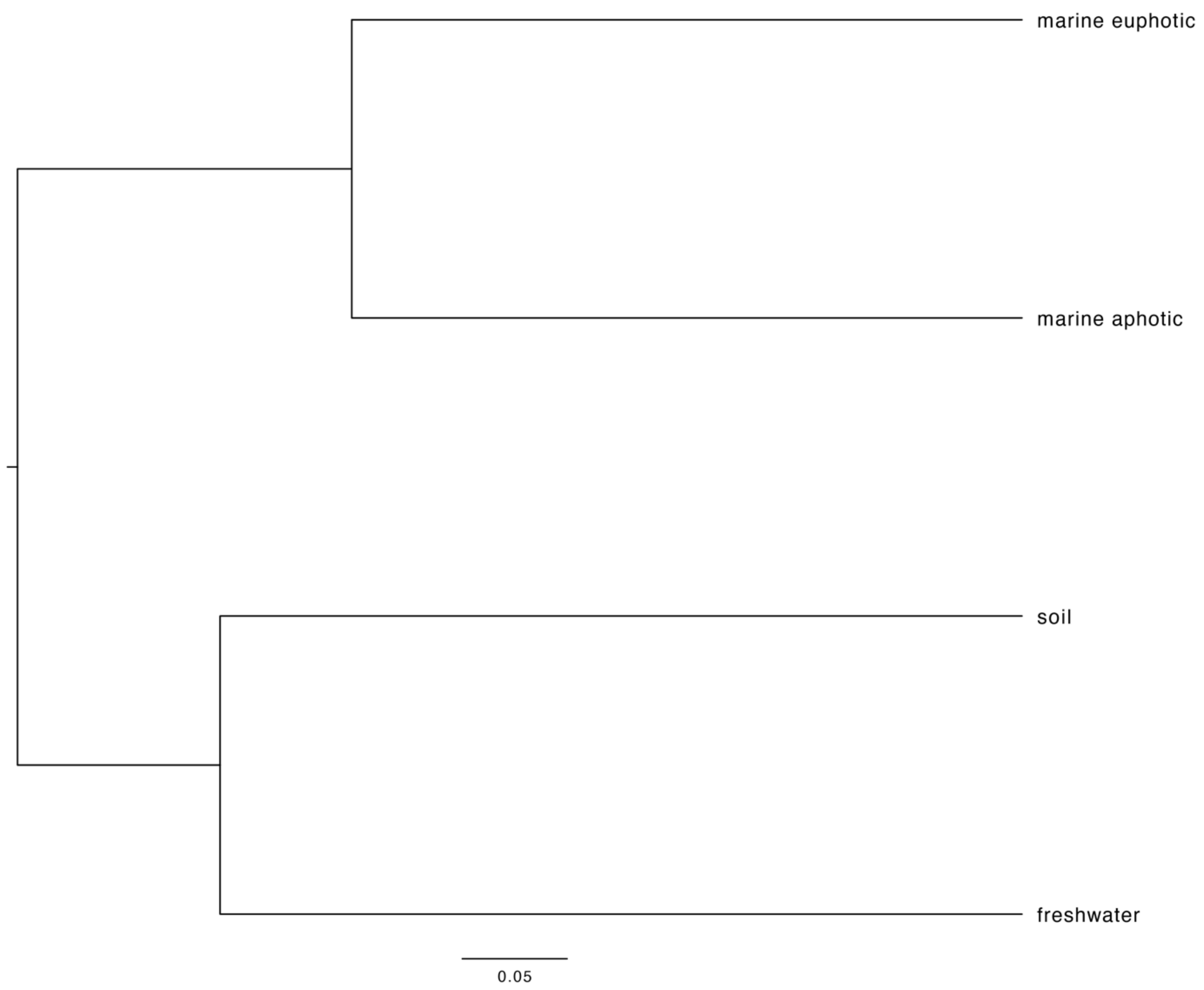

Supplementary Figure 5. UPGMA dendrogram based on Earth mover's distance between placement distributions of marine euphotic, marine aphotic, soil, and freshwater short-read OTUs on the global long-read eukaryotic phylogeny (Supplementary Figure 8). The earth mover's distance in this context refers to the minimum amount of "work" required to shift the placement distribution of one habitat to the distribution of another habitat.

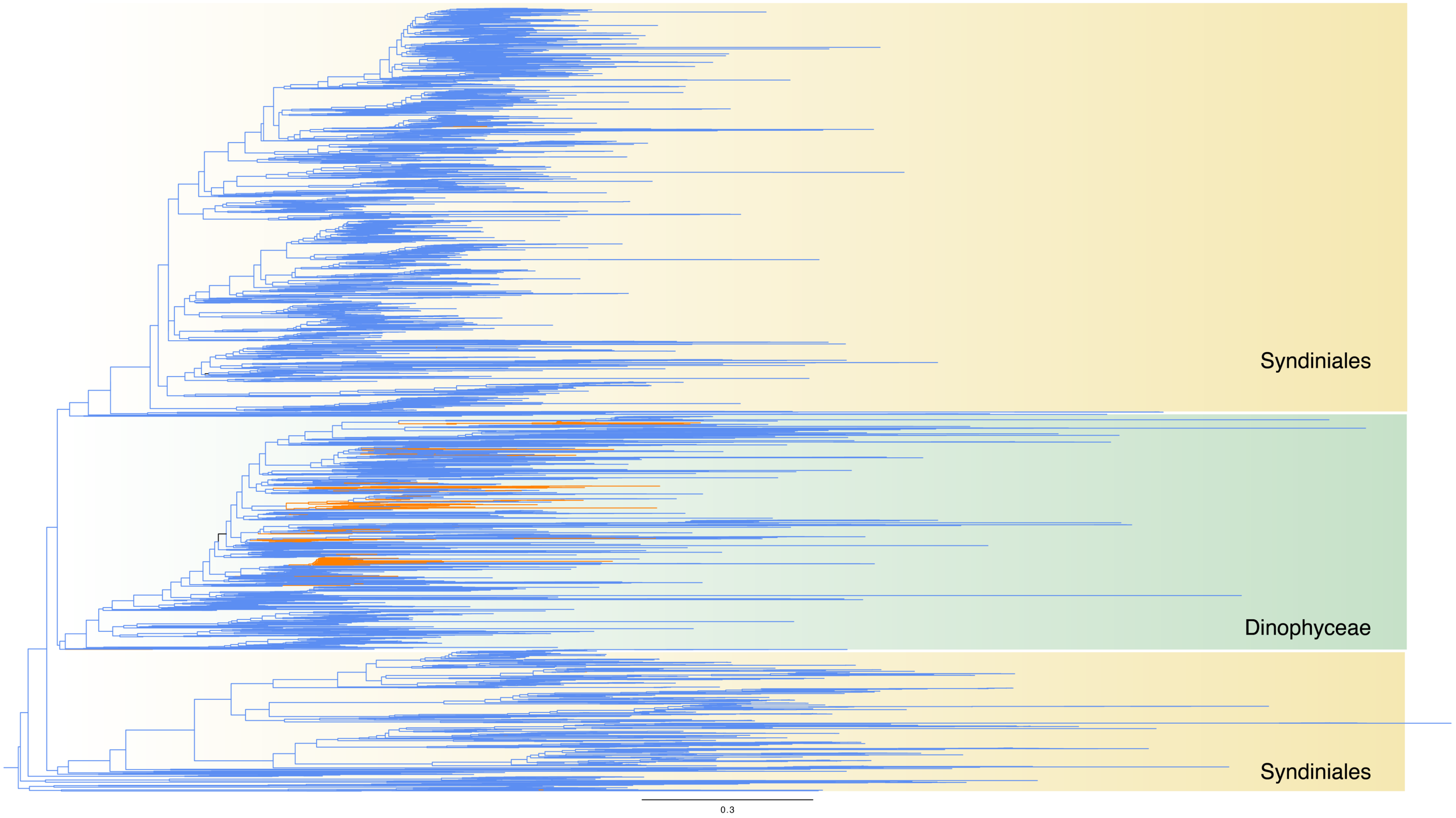

Supplementary Figure 6. Clade-specific (long-read + short-read) phylogeny of dinoflagellates (7154 taxa) (one phylogeny was randomly chosen for display from the set of 100 trees). Branches in blue represent marine clades, while branches in orange represent non-marine clades. This visualisation allows us to make the striking observation that habitat transitions seem to have occurred only in the largely mixotrophic Dinophyceae (also called core dinoflagellates), while almost no habitat transitions have occurred in the parasitic Syndiniales clades.

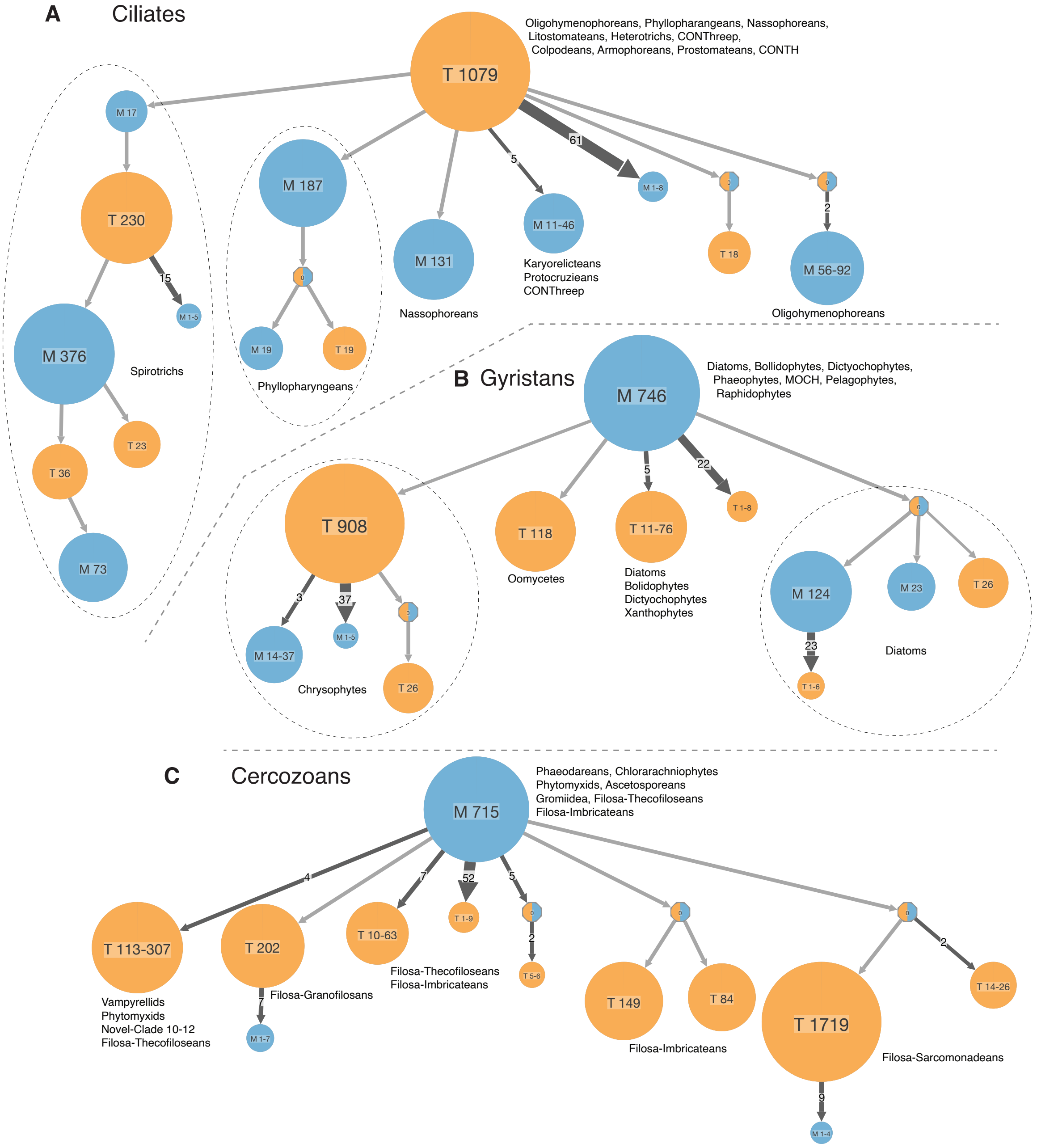

Supplementary Figure 7. Simplified possible ancestral scenarios depicted for (**A**) Ciliates, (**B**) Gyristans, and (**C**) Cercozoans as generated by PASTML using marginal posterior probability approximation (MPPA) with an F-81 like model. For this compressed visualisation, parts of the tree with no state changes are collapsed into circles, with the number in each circle indicating the number of taxa. Orange circles depict non-marine clades, and blue circles depict marine clades. We display the eukaryotic classes represented in each circle, for instance, the first orange circle in ciliates indicates a non-marine origin, with 1079 taxa representing oligohymenophoreans, nassophoreans, colpodeans and many other ciliate classes. Thin grey lines represent a single transition event. For example, Panel **A** shows a single non-marine to marine transition leading to spirotrichs. Thick lines represent multiple transition events with the associated number indicating the number of such events. For example, within spirotrichs (Panel **A**), there were 15 transition events leading to marine clades of sizes one to

five taxa. For the sake of simplification, PASTML displays only major transition events and hides minor ones (further from the root). Therefore certain transition events are not depicted, such as transitions from marine to non-marine environments in chrysophytes, and such as transitions from non-marine to marine environments in vampyrellids.

This figure depicts the high number of serial transitions in spirotrich ciliates for which we can observe repeated re-colonizations of marine and non-marine habitats. Also depicted are a high number of independent transitions within diatoms, chryosphytes (in Panel **B**), and thecofiloseans and imbricateans (in Panel **C**) in accordance with Supplementary Figure 12.

This figure also shows transition events which led to the establishment of several key-deep branching lineages. This pattern is best exemplified by cercozoans, where one putative transition from marine to non-marine environments led to the diversification of vampyrellids, a group of predatory amoebas which hunt algae (Panel **C**); and another putative transition in the same direction led to the largely uncharacterized Novel-10-12 clade[3] which is commonly found in freshwaters.

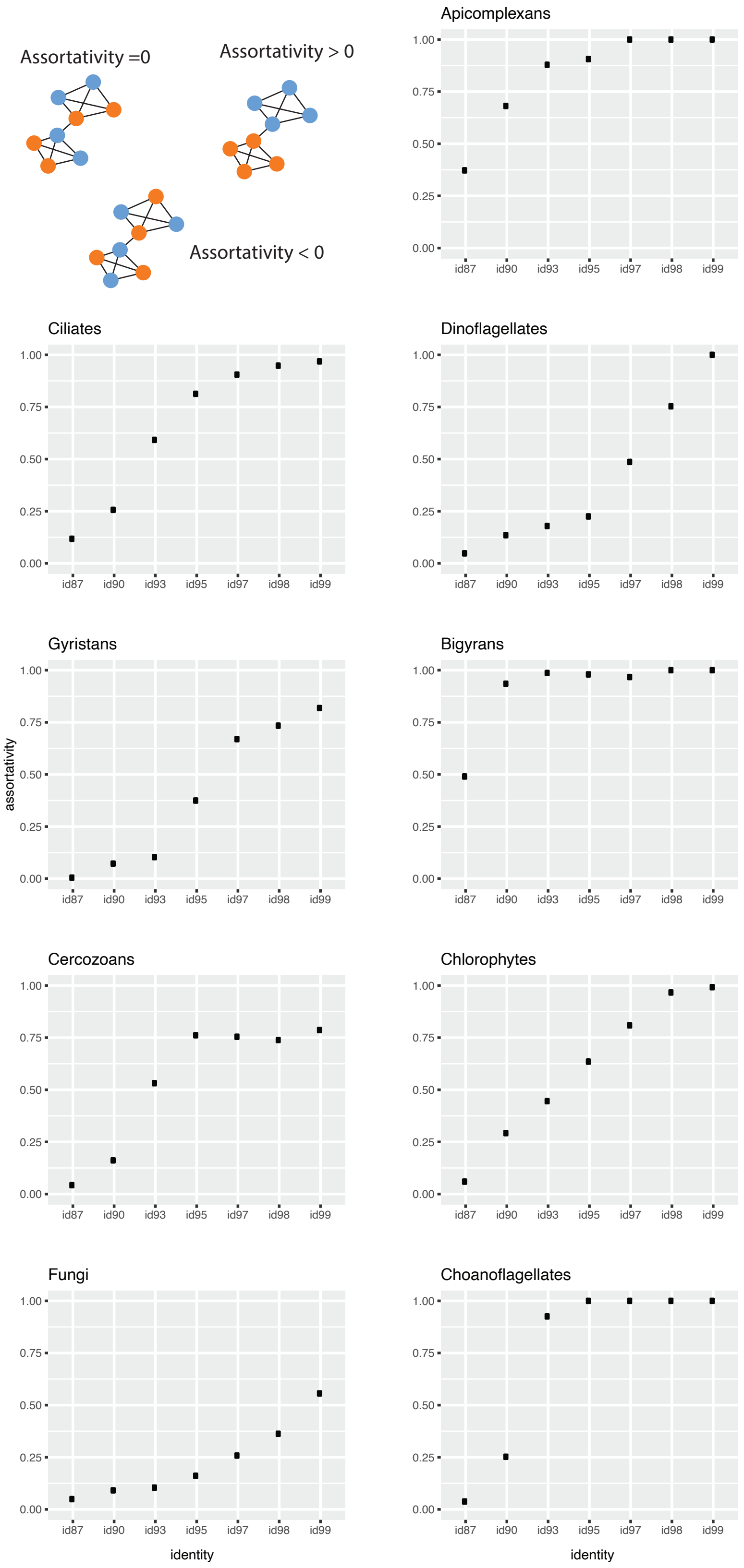

Supplementary Figure 8. Sequence similarity network analyses on PacBio sequences. 18S sequences were clustered at different similarity thresholds to produce networks. A simple representation is shown

in the top right panel, where each node represents a sequence, and each edge represents the connection between nodes. The assortativity of marine and non-marine sequences was measured, which measures whether nodes with the same attribute tend to connect with each other more than random or not. When assortativity = 0, that indicates, in this case, that marine and non-marine nodes connect to each other randomly. When assortativity tends towards 1, that means that marine and non-marine nodes tend to preferentially connect to nodes from the same habitat. With these analyses, we can see that fungi have the lowest assortativity values. This can also be taken to mean that fungal transitions have occurred very recently, so that ribosomal sequences have not had enough time to evolve since the transition.

A

	No. of OTUs	Subsample soil (% of OTUs excluded) 5%	10%	20%	30%	50%	70%
**Soil**	11,935	11,358 - 11,293	10,795 - 10,676	9,676 - 9,470	8,404 - 8,263	6,093 - 5,904	3,717 - 3526
**Freshwater**	3,788	3,788	3,788	3,788	3,788	3,788	3,788
**Marine euphotic**	9,977	9,977	9,977	9,977	9,977	9,977	9,977
**Marine aphotic**	2,518	2,518	2,518	2,518	2,518	2,518	2,518

	No. of OTUs	Subsample marine euphotic (% of OTUs excluded) 5%	10%	20%	30%	50%	70%
**Soil**	11,935	11,935	11,935	11,935	11,935	11,935	11,935
**Freshwater**	3,788	3,788	3,788	3,788	3,788	3,788	3,788
**Marine euphotic**	9,977	9,486 - 9,436	9,064 - 8,940	8,039 - 7,925	7,029 - 6,922	5,035 - 4,898	3,036 - 2943
**Marine aphotic**	2,518	2,518	2,518	2,518	2,518	2,518	2,518

	No. of OTUs	Subsample soil + marine euphotic (% of OTUs excluded) 5%	10%	20%	30%	50%	70%
**Soil**	11,935	11,419 - 11,279	10,798 - 10,714	9,612 - 9,506	8,444 - 8,329	6,036 - 5,844	3,643 - 3,515
**Freshwater**	3,788	3,788	3,788	3,788	3,788	3,788	3,788
**Marine euphotic**	9,977	9,507 - 9,464	9,047 - 8,977	8,059 - 7,911	7,025 - 6,953	5023 - 4944	3,005 - 2959
**Marine aphotic**	2,518	2,518	2,518	2,518	2,518	2,518	2,518

B

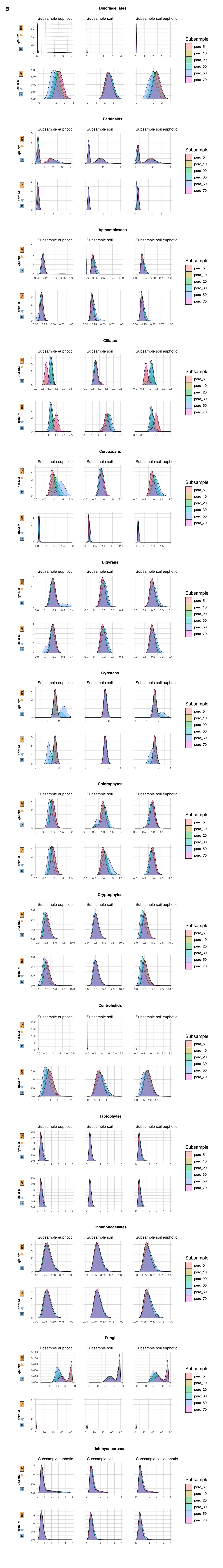

Supplementary Figure 9. (On previous page) Sensitivity test to assess how transition rates are impacted by sampling efforts. The short-read metabarcoding surveys included in this study have uneven sampling efforts in the four habitats considered, with the highest numbers of samples and greatest sequencing depth for marine euphotic and soil environments (see also Supplementary Table 4). As a result, marine euphotic and soil habitats had considerably more OTUs as compared to freshwater and marine aphotic habitats. To assess whether this imbalance in sampling effort had any impact on the transition rates inferred, we carried out a sensitivity test where we increasingly subsampled (1) soil, (2) marine euphotic, and (3) both soil and marine euphotic OTUs in a random fashion, and re-estimated transition rates for each clade. For each subsampling level (from 5% to 70% of the OTUs removed), we replicated the test five times.

(A) Table showing the number of OTUs retained for each subsampling level. The number of OTUs retained varied slightly in each replicate as different random seeds were used to subsample the fasta files using SeqKit[4].

(B) Density plots of transition rates (q_{M-NM} and q_{NM-M}) for each clade across different subsets of taxa. Most clades show the same patterns of directionality in all cases, even if the values are slightly shifted, e.g. in dinoflagellates and cercozoans. This result indicates that transition rates are largely robust to sampling efforts. Two clades show an exception to this result: ciliates and gyristans. Subsampling marine euphotic taxa (20-50% of taxa) results in q_{M-NM} and q_{NM-M} being symmetrical in ciliates, and $q_{M-NM} > q_{NM-M}$ in gyristans.

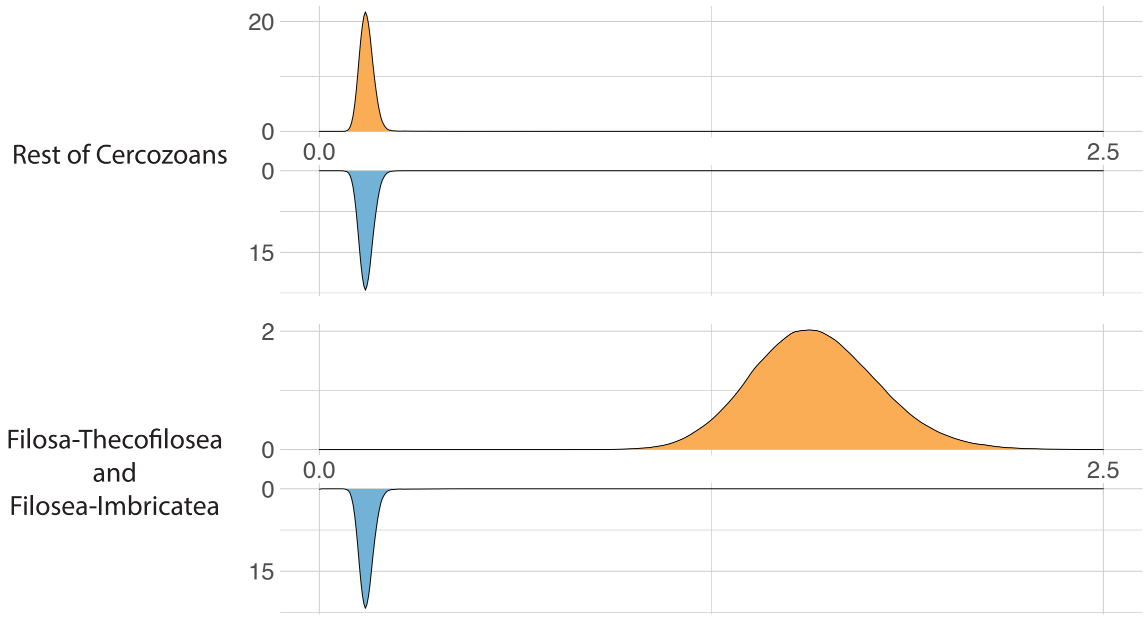

Supplementary Figure 10. Habitat transition rates vary within Cercozoans. Here, we used a heterogeneous model of habitat evolution such that q_{M-NM} (in orange) and q_{NM-M} (in blue) were estimated separately for Filosa-Thecofilosea + Filosa-Imbricatea, and the rest of Cercozoans. Filosa-Thecofilosea + Filosa-Imbricatea was selected based on visual inspection of the Cercozoan phylogenies (where non-marine and marine lineages were more interspersed and not as distinct).

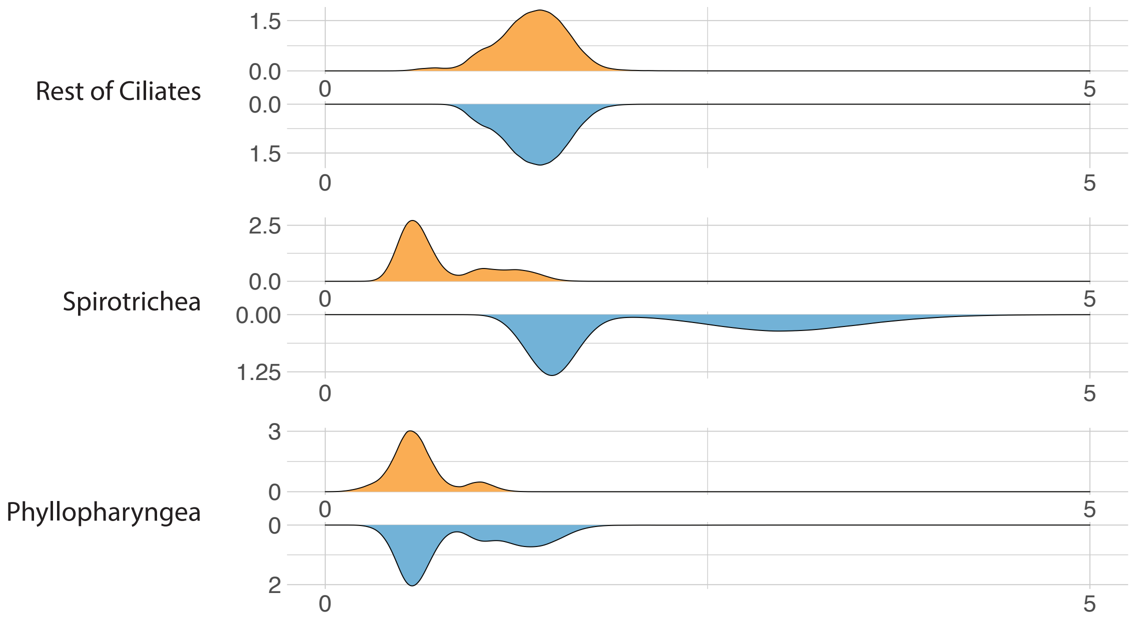

Supplementary Figure 11. Habitat transition rates vary within Ciliates. Here, we used a heterogeneous model of habitat evolution such that q_{M-NM} (in orange) and q_{NM-M} (in blue) were estimated separately for Spirotrichea, Phyllopharyngea and the rest of Ciliates. Spirotrichea and Phyllopharyngea were selected as they showed multiple putative serial colonization events (see Supplementary Figure 14).

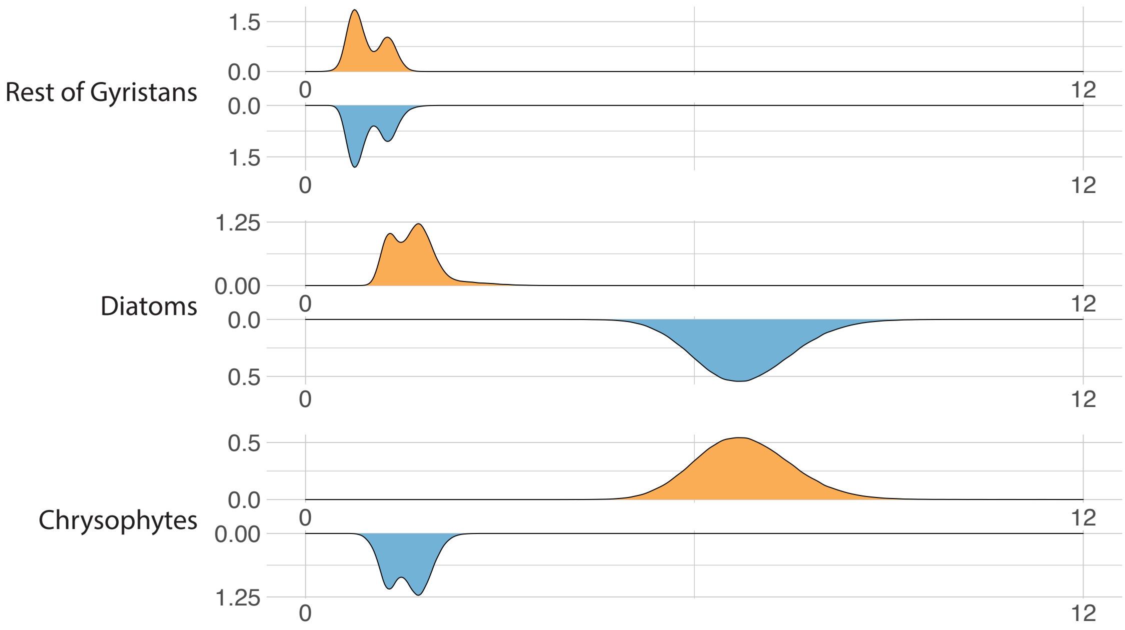

Supplementary Figure 12. Habitat transition rates vary within Gyristans. Here, we used a heterogeneous model of habitat evolution such that q_{M-NM} (in orange) and q_{NM-M} (in blue) were estimated separately for Diatoms, Chrysophytes and the rest of Gyristans. Diatoms and Chrysophytes seemed to have less distinct marine and non-marine lineages based on visual inspection of the Gyristan phylogenies, hence we chose to characterize these clades separately.

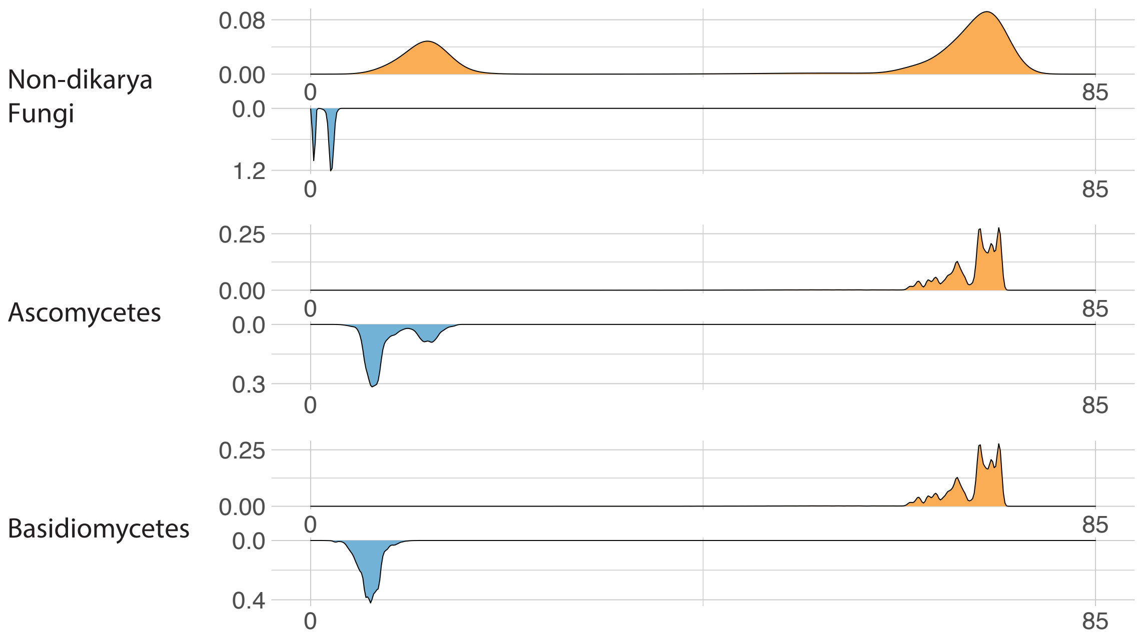

Supplementary Figure 13. Habitat transition rates vary within Fungi. Here, we used a heterogeneous model of habitat evolution such that q_{M-NM} (in orange) and q_{NM-M} (in blue) were estimated separately for Ascomycetes, Basidiomycetes, and non-Dikarya Fungi. Visual inspection of fungal phylogenies indicated more transition events in Dikarya as compared to non-Dikarya.

A

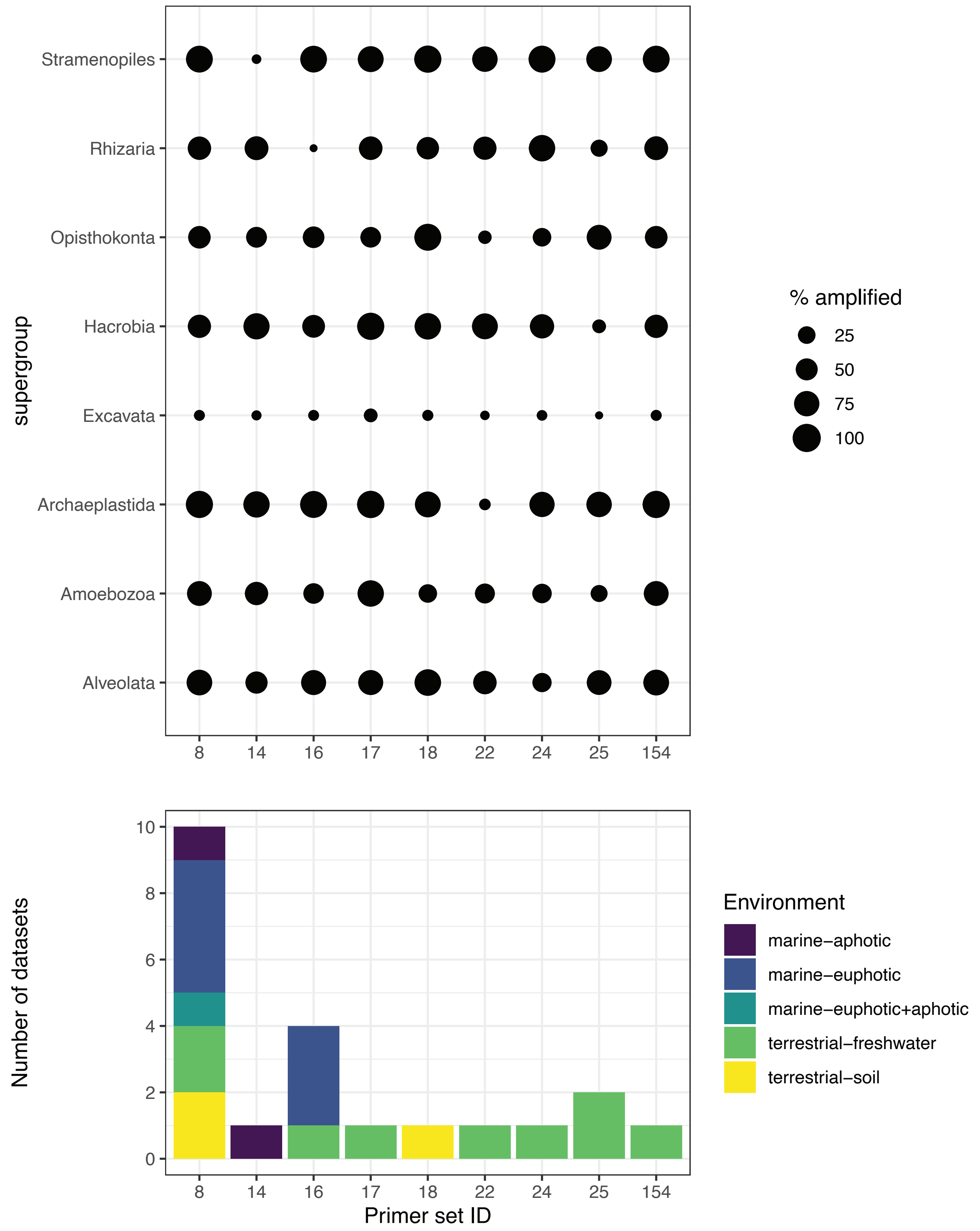
Stramenopiles
Rhizaria
Opisthokonta
Hacrobia
Excavata
Archaeplastida
Amoebozoa
Alveolata
supergroup
% amplified
25
50
75
100
8
14
16
17
18
22
24
25
154
Number of datasets
0
2
4
6
8
10
Environment
marine–aphotic
marine–euphotic
marine–euphotic+aphotic
terrestrial–freshwater
terrestrial–soil
Primer set ID

B

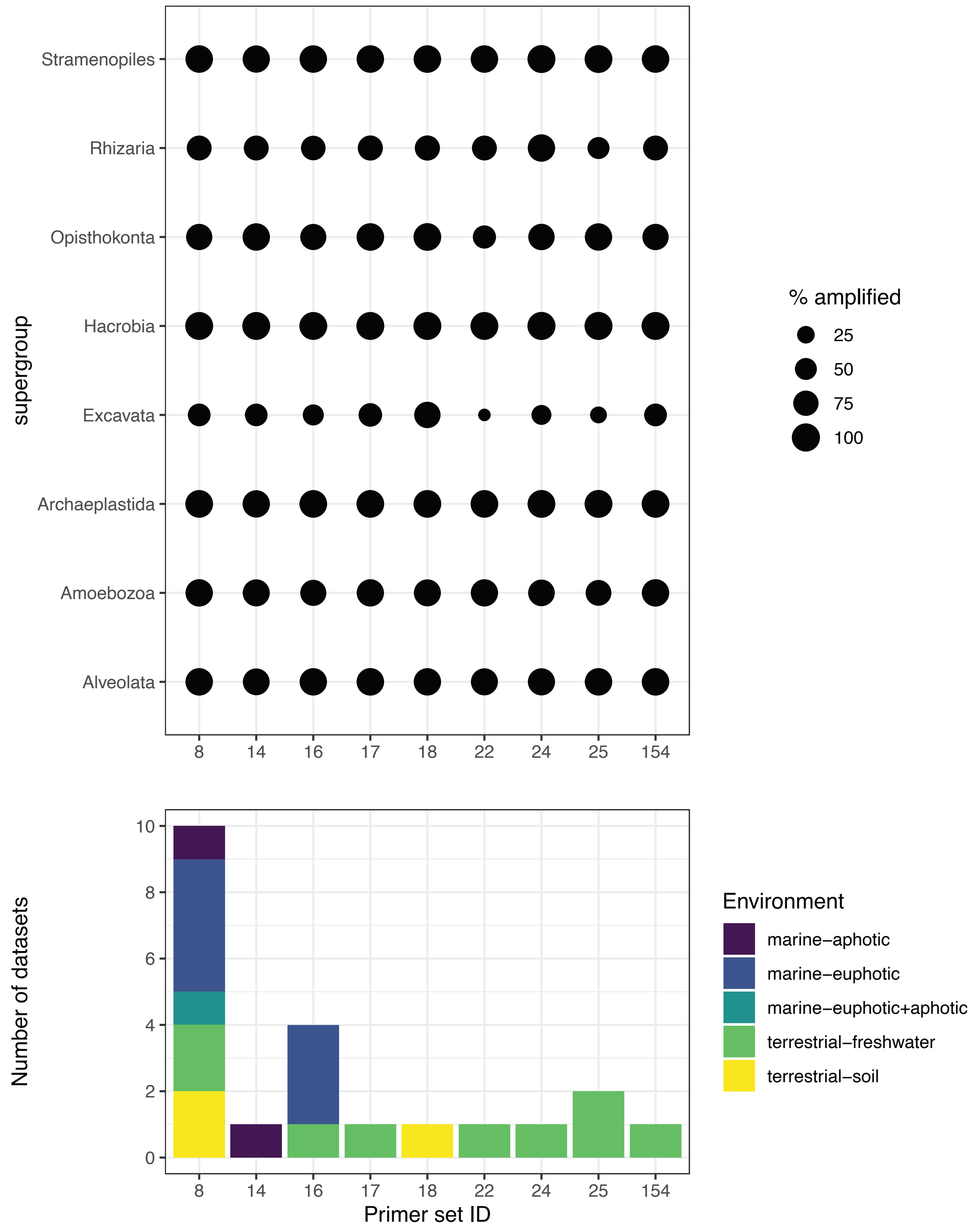
Stramenopiles
Rhizaria
Opisthokonta
Hacrobia
Excavata
Archaeplastida
Amoebozoa
Alveolata
supergroup
% amplified
25
50
75
100
8
14
16
17
18
22
24
25
154
Number of datasets
0
2
4
6
8
10
Environment
marine–aphotic
marine–euphotic
marine–euphotic+aphotic
terrestrial–freshwater
terrestrial–soil
Primer set ID

Supplementary Figure 14. (On previous page). *In-silico* analysis of the nine primer pairs that generated the short-read data used in this study. Primer set IDs correspond to the ID numbers on the web application of PR^2 primers[5]. (**A**) Primer set specificity when allowing no mismatches. (**B**) Primer set specificity when allowing four mismatches (which matches the default setting for the error-tolerance in Cutadapt[6], used for trimming primers, assuming primer lengths of 20 base pairs). The results show that no single habitat has been surveyed with primer pairs all biased against the same eukaryotic clade. We can therefore by reasonably confident that we do not detect (false positive) transition events due to eukaryotic lineages being detected in one environment but not in the other due to primer bias.

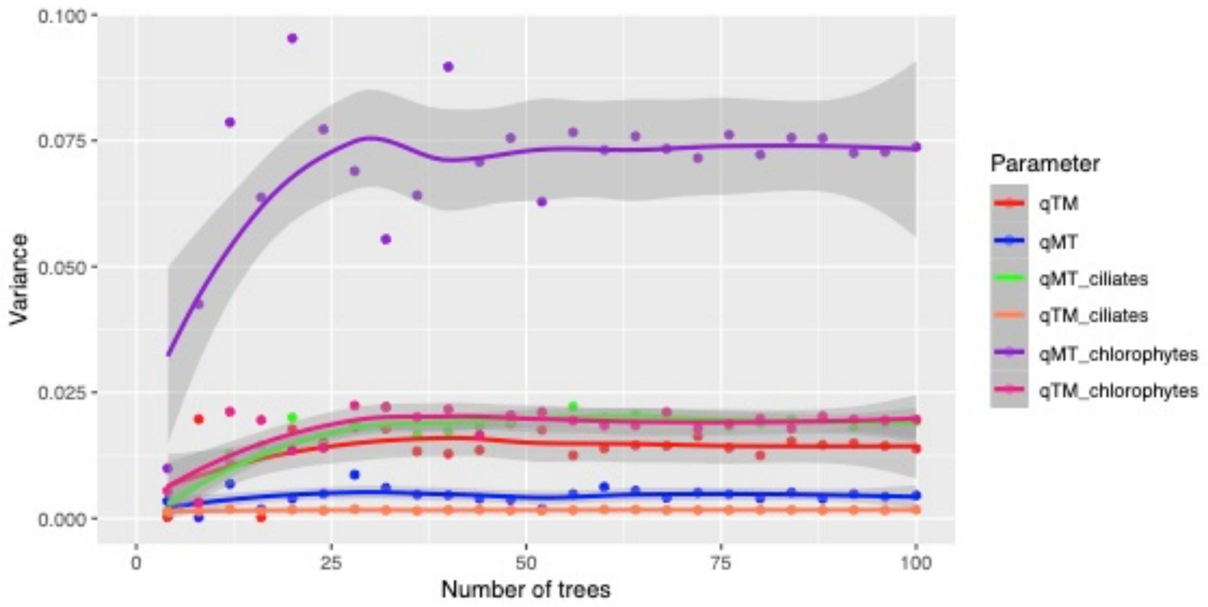

Supplementary Figure 15. The saturation of variance values of transition rate parameters as a function of the number of ML trees. Trees were added in randomized order, in groups of four. Only six parameters are shown here for clarity, but the rest also show similar trends. Lines indicate locally estimated scatterplot smoothing (loess) curves with the grey bars representing 95% confidence intervals.

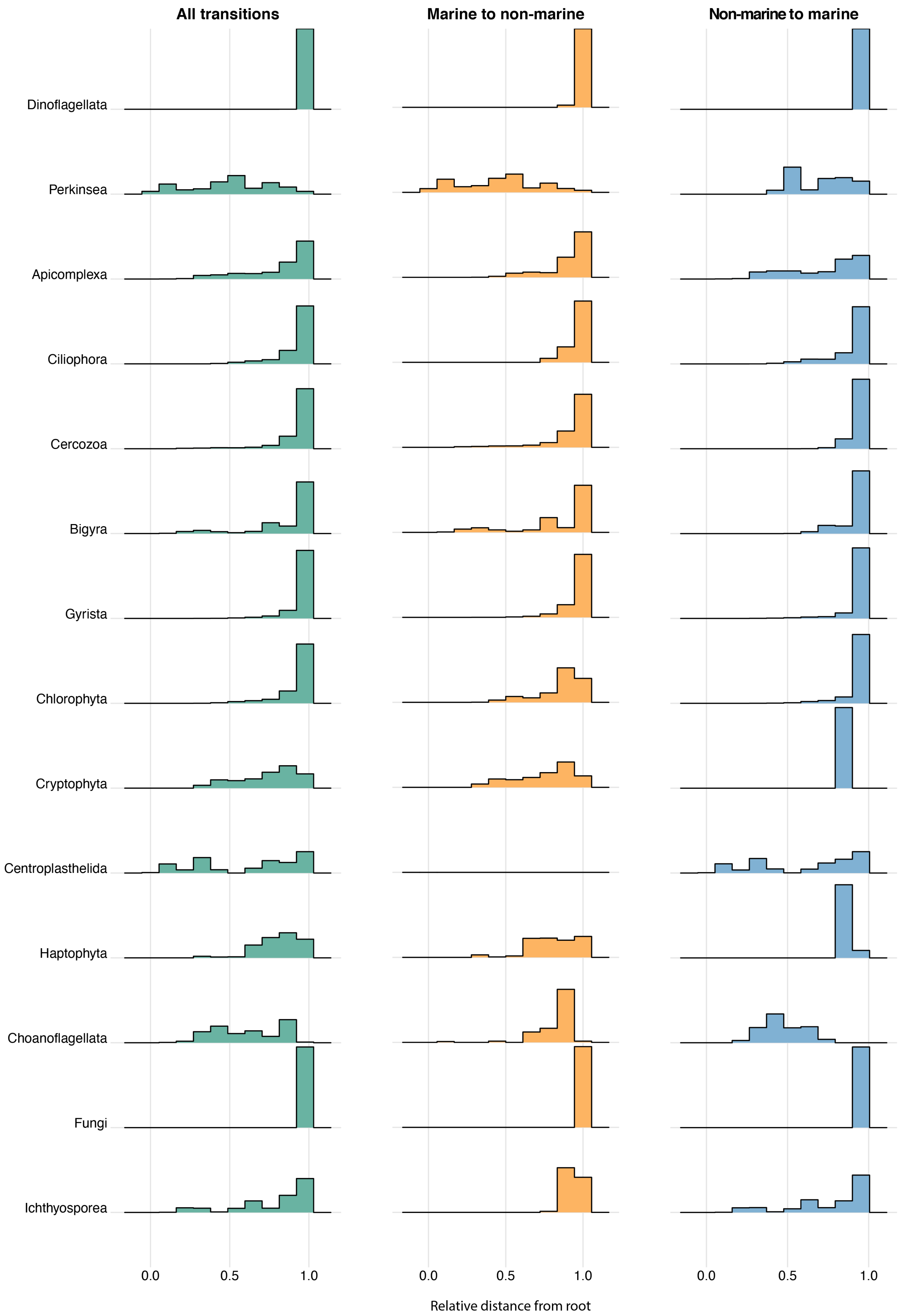

Supplementary Figure 16. Ridgeline histogram plots showing relative timing of transitions. Phylogenies were converted to relative chronograms (root age = 1) with TreePL[7]. These results show

similar patterns as the analyses with Pathd8: (i) most transitions occurred in recent time, and (ii) perkinsids, choanoflagellates, and centrohelids have geologically older transitions. We opted to display the Pathd8 analyses in the main text as TreePl suggests that all transitions in dinoflagellates occurred 100 mya or later (assuming that the clade arose 1 billion years ago[8]). This result is in conflict with studies that have suggested transitions as early as 140 mya[9] and possibly older[10,11].

Supplementary Note 1. How many ML trees are enough?

It is important to take phylogenetic uncertainty into account when performing ancestral state reconstruction analyses. We assessed whether 100 ML trees were enough to represent the plausible set of topologies given the dataset. This was done in two ways.

1) Robinson-Foulds distance

We calculated relative pairwise Robinson-Foulds (RF) distances in raxml-ng (using the command --rf-dist), where an RF-distance of 0 indicates identical topologies, and 1 indicates that no bipartitions are shared. As we had only constrained 60 internal branches out of the possible 16818 branches, the upper bound on the possible pairwise RF-distance of two trees (T1 and T2) was close to 1 (calculation shown below).

$$\text{Maximum RF distance} = \frac{total\ number\ of\ possible\ unshared\ bipartitions\ in\ T1\ and\ T2}{total\ number\ of\ bipartitions\ in\ both\ trees}$$

$$= \frac{(16818-60)(2)}{(16818)(2)} = 0.996$$

Our calculations showed that the 100 ML trees represented 100 unique topologies with an average relative RF distance of 0.0675, indicating that the trees are topologically very similar on average.

2) Bootstrapping test

We performed the auto MRE-based bootstrapping test in raxml-ng using a cutoff value of 0.05 (--bsconverge --bs-cutoff 0.05). The algorithm is typically employed to assess how many bootstrap replicates are enough by considering the variance in the tree set[12]. Briefly, the tree set is split into two sub-sets with 1000 permutations, and the weighted RF distance is calculated between the majority-rule consensus trees of the two sub-sets. We performed the auto MRE based bootstrapping test on our set of 100 ML trees and found that convergence was reached after 50 trees.

Supplementary Note 2. Habitat transition rates in Fungi

(i) Heterogeneity in fungal transition rates

While our analyses show an extremely high habitat transition rate for fungi (~90 expected transitions per substitution/site), it is important to note that this number hides important variation in the transition rates of different fungal groups. Consistent with previous literature[13,14], only selected clades had marine representatives (Supp. Note2 Fig 1). These groups include Pezizomycotina (in particular Dothideomycetes, Sordariomycetes), Saccharomycotina, Ustilaginomycotina, and Chytridiomycota. On the other hand, groups like Glomeromycota and Glomeromycotina had few or no marine representatives (Supp. Note2 Fig 2).

When visually inspecting phylogenies, we observed that marine and non-marine sequences in groups such as Pezizomycotina are extremely closely related (with little to no genetic distance between them based on the 18S and 28S genes; Supp. Note2 Fig 1), which is consistent with the high transition rates estimated by our analyses. These results are also consistent with previous literature on the subject[13,14]. For example, the genus *Malassezia*, which is well-known to be linked to skin conditions such as dandruff, is also found in a range of marine habitats, from coral reefs to deep sea vents[15]. In fact, phylogenies of this genus show that marine and non-marine sequences are not monophyletic, but are interspersed within the phylogeny, suggesting multiple transition events within this genus alone. Given that we clustered our sequences at 97% similarity, it is likely that the number of transition events in Fungi (as shown in Figure 2c-d in the manuscript) are underestimates, since we are unable to detect very recent transition events."

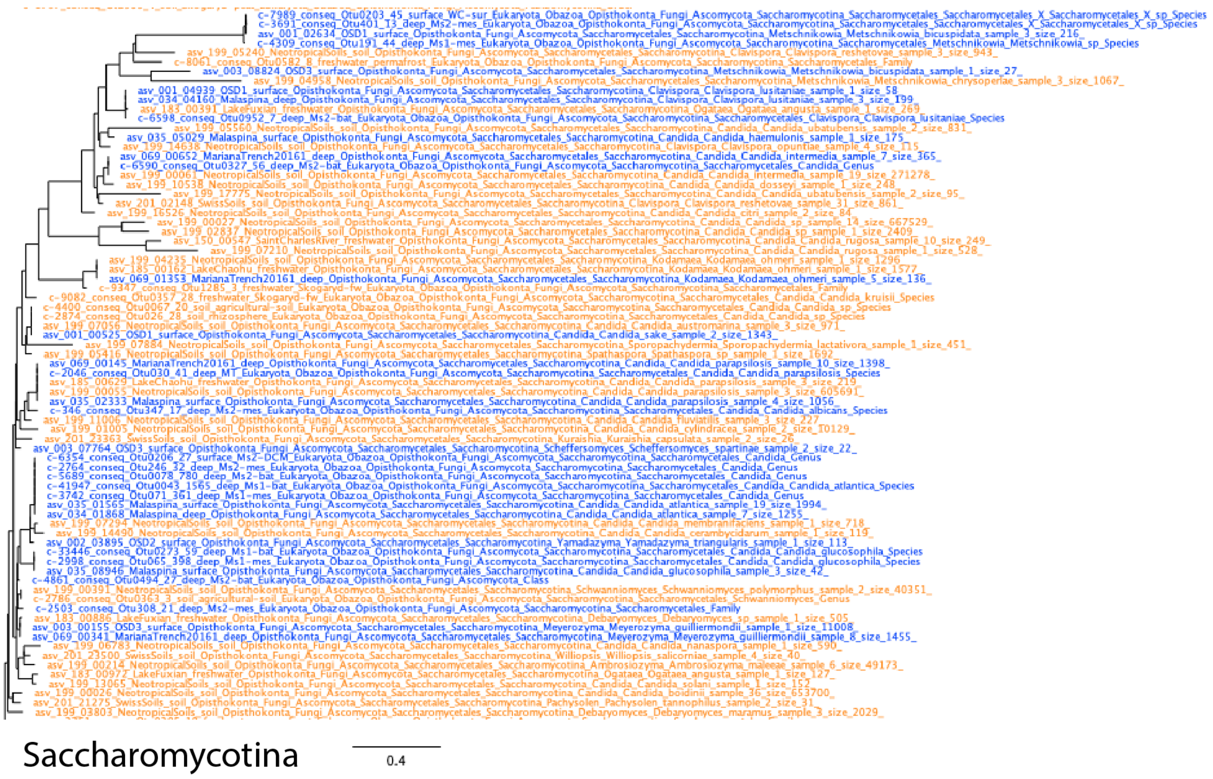

Supplementary Note2 Fig 1. Section of a phylogeny from Saccharomycotina which includes many ascomycete yeasta. Taxa in orange are non-marine, while taxa in blue are marine.

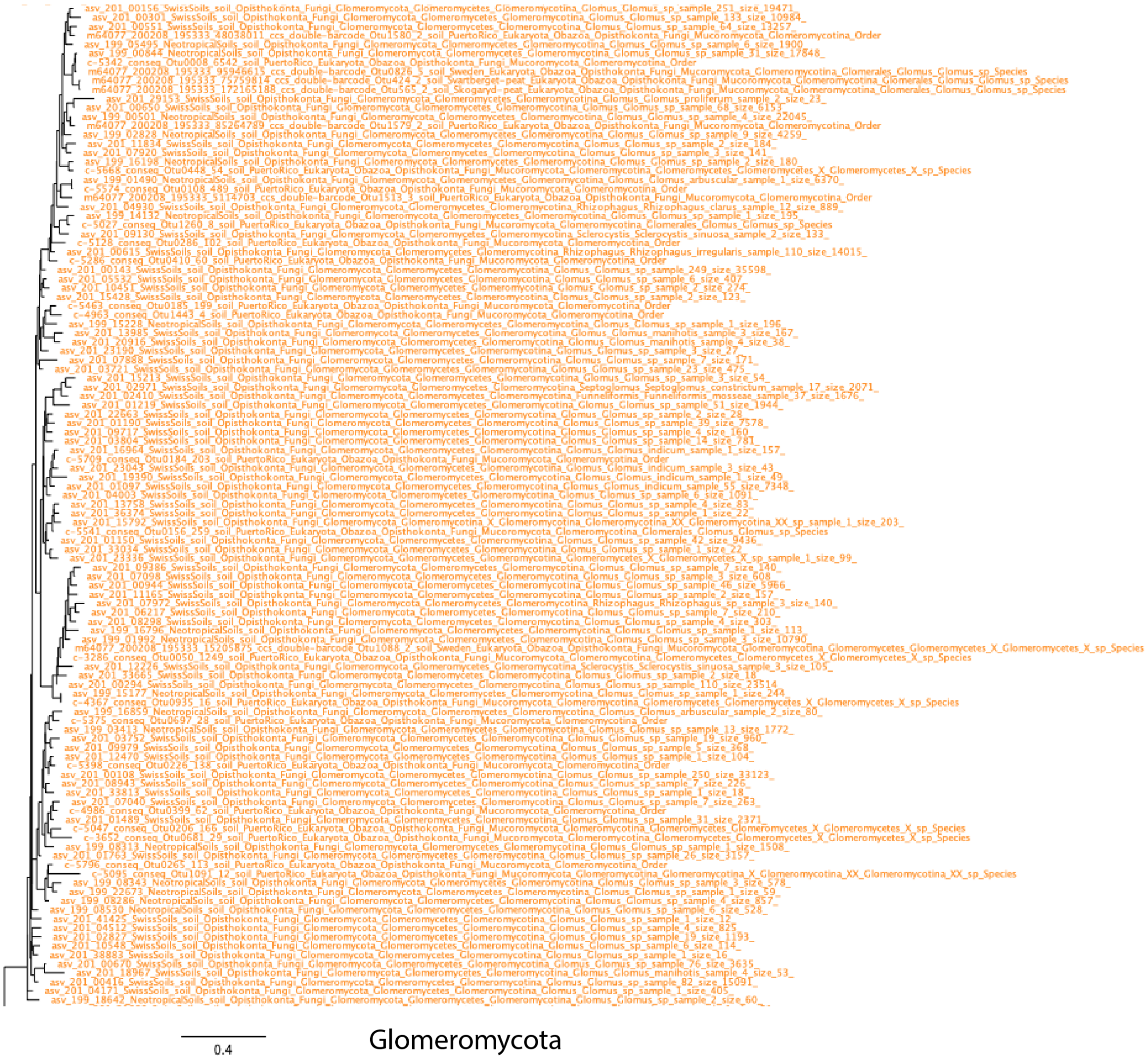

Supplementary Note2 Fig 2. Section of a phylogeny of Glomeromycota with non-marine-only taxa.

(ii) Fungal spores may be able to resist difficult conditions

Fungal spores may be able to persist in conditions in which the fungus itself is not able to live. For instance, spores of soil fungi may end up in seas, and their DNA can then be picked up in metabarcoding surveys. To assess the possibility that the ubiquity of fungal spores leads to an overestimation of transition rates, we examined how marine OTUs were distributed in our fungal phylogenies, focusing in particular on the short-read OTUs. If fungal spores were indeed ubiquitous, we should see marine OTUs distributed indiscriminately across the fungal phylogeny. However, we observed that 359/414 (i.e. 86%) of marine OTUs belonged to clades previously known to be living in marine water column and sediment samples[13,14] (Supp. Note2 Table 1). This result indicates that fungal spores may not occur as ubiquitously as presumed (and/or our stringent filtering of sequences removes most “contaminants”), and therefore should not influence transition rate analyses so much.

Supplementary Note2 Table 1. Distribution of OTUs among fungal clades based on a randomly selected phylogeny of long- and short-read sequences. All clades listed, except the last row, are known to have marine representatives.

	Short-read OTUs	Long-read OTUs	All OTUs
**Agaricomycotina**	15	17	32
**Ustilaginomycotina**	14	21	35
**Pezizomycotina**	32	62	94
**Saccharomycotina**	32	20	52
**Puccinomycotina**	23	21	44
**Chytridiomycota**	78	2	80
**Cryptomycota**	18	4	22
**Sequences interspersed among other clades**	46	9	55

(iii) Evidence indicating that marine fungi are metabolically active

Several studies provide further evidence indicating that marine fungi are true marine residents, contributing to biogeochemical cycles. First, RNA sequencing of the 18S gene recovers fungi as one of the most dominant groups in marine sediment communities[16,17], indicating that fungi are metabolically active, and not inactive resting spores. Second, marine fungal communities have been found to be structured by habitat, temperature, salinity, and other environmental factors[18–20]. For instance, saprotrophic fungi have low abundance and diversity in upper marine water column environments (which are often nutrient poor and dominated by free floating or swimming photosynthetic and grazing protists)[14], but dominate sediments and marine snow particles in the bathypelagic[17,21,22] where they presumably have increased niche availability. On the other hand, chytrids and cryptophytes infecting eukaryotic algae are more dominant in colder, pelagic habitats[23,24]. Third, several studies have shown the role of marine fungi by measuring their extracellular enzyme activity[25], the amount of carbon they assimilate from phytoplankton[26], and assessing active metabolic pathways using metatranscriptomics[27,28].

Altogether, these results indicate that indeed Fungi have the highest habitat transition rates among eukaryotes (although we acknowledge that some marine fungal sequences might be spores sourced from soils/freshwater), which represents an interesting hypothesis to be further tested.

Supplementary Note 3. Estimate of ancestral habitats of eukaryotic groups with insufficient PacBio data.

Collodictyonids. *Collodictyon triciliatum* was first isolated and described from freshwater habitats[29–31]. An environmental survey using group specific primers revealed a global distribution that was limited to freshwater environments[32]. No collodictyonid has been found in marine environments, therefore a non-marine origin seems more likely.

Metamonads. Mantamonads are gliding flagellates that have thus far only been found in benthic marine habitats[33,34].

Pluriformeans. This group currently consists of two genera: *Corallochytrium* which is a marine organism associated with corals, and *Syssomonas* which is found in freshwater. The ancestral habitat of this group is thus ambiguous[35].

Ancoracystids. The only described organism from this group, *Ancoracysta twista*, was isolated from the surface of a marine coral[36].

Glaucophytes. Almost all glaucophyte algae inhabit freshwater environments, making a non-marine origin more likely[37–39].

Rhodophytes. While several rhodophytes are found in freshwater lakes and even soils, the vast majority of rhodophytes are marine, and ancestral state reconstruction studies indicate a marine origin[38,39].

Rhodelphids. Two species have been described from this group thus far: *Rhodelphis marinus* which was obtained from marine coral sand, and *Rhodelphis limneticus* which was obtained from a freshwater lake[40]. The ancestral habitat of the group is therefore ambiguous.

Supplementary Table 1. Collection details of the 21 samples sequenced with PacBio Sequel II for this study. All raw sequencing data can be accessed at ENA under accession PRJEB45931 (this study) and PRJEB25197 (sequenced in [41]). SITES = Swedish Infrastructure for Ecosystem Science. SMHI = Swedish Meteorological and Hydrological Institute.

No.	Sample code	Sample type, level 1	Sample type, level 2	Sampling date (yyyy-mm-dd)	Sample collected by	No. of sites pooled	Country/Region	Sampling Location	Comments	Size fraction	Lat	Long	Depth	Reference for samples	Sequencing instrument	No. of demultiplexed reads	No. of processed reads	No. of OTUs
1	Erken_fw	non-marine	freshwater	2019-10-09	SITES	1	Sweden	Lake Erken, Erken research station	Hemi-boreal, agricultural landscape	200-0.2 µm	59.83	18.63	0-20 m	This study	Sequel II	564,504	320,773	768
2	Skogaryd_fw	non-marine	freshwater	2019-10-17	SITES	1	Sweden	Lake Ersjön, Skogaryd research station	Hemi-boreal, forest landscape	200-0.2 µm	58.37	12.16	0.5, 2, 4 m	This study	Sequel II	699,716	422,836	1688
3	Svartberget_fw	non-marine	freshwater	2019-11-05	SITES	1	Sweden	Lake Stortjärn, Svartberget research station	Boreal, forest landscape	200-0.2 µm	64.24	19.76	0.5, 3.5 m	This study	Sequel II	627,565	373,152	496
4	Permafrost_fw	non-marine	freshwater	2014-08	S. Peura, K. Einarsdottir, M. Wauthy	10	Canada	Nunavik, Quebec	Multiple permafrost thaw ponds	> 0.2 µm	55.22	-77.69	0-1.7 m	[42]	Sequel II	456,683	256,770	1324
5	Erken_sed_fw	non-marine	freshwater	2019-10-09	SITES	4	Sweden	Lake Erken, Erken research station	Sediment core of depth 0-5 cm	No size selection	59.83	18.63	0-5 cm	This study	Sequel II	519,814	337,656	1391
6	Skogaryd_soil	non-marine	soil	2019-10-17	SITES	4	Sweden	Skogaryd Mire, Skogaryd research station	Composite sample from wet and dry areas	No size selection	58.37	12.16	top soil	This study	Sequel II	301,806	90,907	593
7	Svartberget_soil	non-marine	soil	2019-11-05	SITES	3	Sweden	Kallkäls Mire, Svartberget research station		No size selection	64.24	19.76	top soil	This study	Sequel II	241,901	133,266	763
8	PuertoRico_soil	non-marine	soil	2013-12	H. Urbina	6	Puerto Rico	El Yunque, Puerto Rico	Montane wet forest	No size selection	18.29	-65.78	top soil	[43]	Sequel II	770,855	384,698	441
9	Sweden_soil	non-marine	soil	2013-10-15	H. Urbina	6	Sweden	Ivantjärnsheden field station, Jädraås	Pine forest	No size selection	60.49	16.3	top soil	[44]	Sequel II	950,141	519,140	1184
10	agricultural_soil	non-marine	soil	2008-03-04	P. Gosling, G. Bending	14	UK	Set aside arable sites, UK	Sequenced in previous study	No size selection	51.21	1.23	top soil	[41]	Sequel	36,339	10,755	595
11	rhizosphere_soil	non-marine	soil	2015-03	S. Hilton	33	UK	25 commercial farms in the UK	Sequenced on Sequel I	No size selection	52.2	-0.81	top soil	[41]	Sequel	28,347	9,013	222
12	Tibet_soil	non-marine	soil	06/08-2011	S. Geisen, J. Zhang	9	Tibet	Mix of alpine meadows, shrubs, and forest	Sequenced in previous study	No size selection	29.36	94.43	top soil	[41]	Sequel	48,676	4,315	183
13	WC_sur	marine	euphotic	2019-08-20	SMHI	1	North Sea	Station Anholt E		200-0.2 µm	56.66	12.11	5 m	This study	Sequel II	1,543,739	964,198	639
14	Ms1_DCM	marine	euphotic	2011-02-17	Malaspina team	2	Indian Ocean	Malaspina station 49	DCM layer	3-0.2 µm	-33.9	37	85 m	[45]	Sequel II	273,073	167,358	1126
15	Ms2_sur	marine	euphotic	2011-03-25	Malaspina team	2	Indian Ocean	Malaspina station 76	Surface	3-0.2 µm	-40.6	142.5	3 m	[45]	Sequel II	149,790	93,932	377
16	Ms2_DCM	marine	euphotic	2011-03-25	Malaspina team	2	Indian Ocean	Malaspina station 76	DCM layer	20-0.2 µm	-40.6	142.5	70 m	[45]	Sequel II	511,880	272,269	754
17	Ms1_mes	marine	aphotic	2011-02-17	Malaspina team	2	Indian Ocean	Malaspina station 49	Mesopelagic	3-0.2 µm	-33.9	37	800 m	[45]	Sequel II	642,804	243,523	540
18	Ms2_mes	marine	aphotic	2011-03-25	Malaspina team	2	Indian Ocean	Malaspina station 76	Mesopelagic	20-0.2 µm	-40.6	142.5	275 m	[45]	Sequel II	656,429	415,020	637
19	Ms1_bat	marine	aphotic	2011-02-17	Malaspina team	2	Indian Ocean	Malaspina station 49	Bathypelagic	20-0.2 µm	-33.9	37	1200 m	[45]	Sequel II	761,055	422,088	1554
20	Ms2_bat	marine	aphotic	2011-03-25	Malaspina team	2	Indian Ocean	Malaspina station 76	Bathypelagic	20-0.2 µm	-40.6	142.5	2800-3300 m	[45]	Sequel II	775,615	483,155	1196
21	MT	marine	aphotic	2016-06	H. Jing, pilots of Jiao Long Hao	1	Pacific Ocean	Mariana Trench	Bathypelagic	3-0.2 µm	11.18	141.98	5900 m	[17]	Sequel II	162,569	84,921	99

Supplementary Table 2. The first five ranks of the PR2-transitions database[46] (upto the class rank; see Materials and Methods for details on how the PR2 database was adapted for this study), and whether corresponding ribosomal DNA sequences were recovered by PacBio sequencing.

Domain	Supergroup	Division	Subdivision	Class	Sequenced by PacBio
Eukaryota	Amoebozoa	Amoebozoa_X	Amoebozoa_XX	Amoebozoa_XXX	Yes
Eukaryota	Amoebozoa	Amoebozoa_X	Amoebozoa_XX	Lobosa-G1	Yes
Eukaryota	Amoebozoa	Discosea	Discosea_X	Centramoebia	Yes
Eukaryota	Amoebozoa	Discosea	Discosea_X	Flabellinia	Yes
Eukaryota	Amoebozoa	Discosea	Discosea_X	Stygamoebida	Yes
Eukaryota	Amoebozoa	Evosea	Evosea_X	Archamoebea	Yes
Eukaryota	Amoebozoa	Evosea	Evosea_X	Eumycetozoa	Yes
Eukaryota	Amoebozoa	Evosea	Evosea_X	Variosea	Yes
Eukaryota	Amoebozoa	Tubulinea	Tubulinea_X	Corycida	-
Eukaryota	Amoebozoa	Tubulinea	Tubulinea_X	Echinamoebida	Yes
Eukaryota	Amoebozoa	Tubulinea	Tubulinea_X	Elardia	Yes
Eukaryota	Amoebozoa	Tubulinea	Tubulinea_X	Tubulinea_XX	Yes
Eukaryota	Archaeplastida	Chlorophyta	Chlorophyta_X	Chlorodendrophyceae	Yes
Eukaryota	Archaeplastida	Chlorophyta	Chlorophyta_X	Chlorophyceae	Yes
Eukaryota	Archaeplastida	Chlorophyta	Chlorophyta_X	Chlorophyta_XX	Yes
Eukaryota	Archaeplastida	Chlorophyta	Chlorophyta_X	Chloropicophyceae	Yes
Eukaryota	Archaeplastida	Chlorophyta	Chlorophyta_X	Mamiellophyceae	Yes
Eukaryota	Archaeplastida	Chlorophyta	Chlorophyta_X	Nephroselmidophyceae	-
Eukaryota	Archaeplastida	Chlorophyta	Chlorophyta_X	Palmophyllophyceae	Yes
Eukaryota	Archaeplastida	Chlorophyta	Chlorophyta_X	Pedinophyceae	Yes
Eukaryota	Archaeplastida	Chlorophyta	Chlorophyta_X	Picocystophyceae	-
Eukaryota	Archaeplastida	Chlorophyta	Chlorophyta_X	Prasino-Clade-9	Yes
Eukaryota	Archaeplastida	Chlorophyta	Chlorophyta_X	Prasino-Clade-VIII	-
Eukaryota	Archaeplastida	Chlorophyta	Chlorophyta_X	Prasino-Clade-V	Yes
Eukaryota	Archaeplastida	Chlorophyta	Chlorophyta_X	Pyramimonadophyceae	Yes
Eukaryota	Archaeplastida	Chlorophyta	Chlorophyta_X	Trebouxiophyceae	Yes
Eukaryota	Archaeplastida	Chlorophyta	Chlorophyta_X	Ulvophyceae	Yes
Eukaryota	Archaeplastida	Glaucophyta	Glaucophyta_X	Glaucocystophyceae	Yes
Eukaryota	Archaeplastida	Rhodelphea	Rhodelphea_X	Rhodelphida	Yes
Eukaryota	Archaeplastida	Rhodophyta	Rhodophyta_X	Bangiophyceae	Yes
Eukaryota	Archaeplastida	Rhodophyta	Rhodophyta_X	Compsopogonophyceae	-

Domain	Supergroup	Division	Subdivision	Class	Sequenced by PacBio
Eukaryota	Archaeplastida	Rhodophyta	Rhodophyta_X	Florideophyceae	-
Eukaryota	Archaeplastida	Rhodophyta	Rhodophyta_X	Porphyridiophyceae	-
Eukaryota	Archaeplastida	Rhodophyta	Rhodophyta_X	Rhodellophyceae	-
Eukaryota	Archaeplastida	Rhodophyta	Rhodophyta_X	Rhodophyta_XX	-
Eukaryota	Archaeplastida	Rhodophyta	Rhodophyta_X	Stylonematophyceae	-
Eukaryota	Archaeplastida	Streptophyta	Streptophyta_X	Charophyceae	-
Eukaryota	Archaeplastida	Streptophyta	Streptophyta_X	Coleochaetophyceae	-
Eukaryota	Archaeplastida	Streptophyta	Streptophyta_X	Embryophyceae	Yes
Eukaryota	Archaeplastida	Streptophyta	Streptophyta_X	Klebsormidiophyceae	Yes
Eukaryota	Archaeplastida	Streptophyta	Streptophyta_X	Mesostigmatophyceae	-
Eukaryota	Archaeplastida	Streptophyta	Streptophyta_X	Streptophyta_XX	Yes
Eukaryota	Archaeplastida	Streptophyta	Streptophyta_X	Zygnemophyceae	Yes
Eukaryota	CRuMs	Collodictyonidae	Collodictyonidae_X	Collodictyonidae_XX	-
Eukaryota	CRuMs	Mantamonadidea	Mantamonadidea_X	Mantamonadida	-
Eukaryota	CRuMs	Rigifilida	Rigifilida_X	Rigifilida_XX	Yes
Eukaryota	Cryptista	Cryptista_X	Cryptista_XX	Cryptista_XXX	-
Eukaryota	Cryptista	Cryptophyta	Cryptophyta_X	Cryptophyceae	Yes
Eukaryota	Cryptista	Kathablepharidacea	Kathablepharida	Kathablepharidea	Yes
Eukaryota	Eukaryota_X	Ancoracystida	Ancoracystida_X	Ancoracystida_XX	-
Eukaryota	Eukaryota_X	Ancyromonadida	Ancyromonadida_X	Ancyromonadida_XX	Yes
Eukaryota	Eukaryota_X	Hemimastigophora	Hemimastigophora_X	Hemimastigophora_XX	Yes
Eukaryota	Eukaryota_X	Picozoa	Picozoa_X	Picozoa_XX	Yes
Eukaryota	Excavata	Discoba	Discoba_X	Euglenozoa	Yes
Eukaryota	Excavata	Discoba	Discoba_X	Heterolobosea	Yes
Eukaryota	Excavata	Discoba	Discoba_X	Jakobida	Yes
Eukaryota	Excavata	Discoba	Discoba_X	Tsukubamonadidae	-
Eukaryota	Excavata	Malawimonadidae	Malawimonadidae_X	Malawimonadidae_XX	Yes
Eukaryota	Excavata	Metamonada	Metamonada_X	Fornicata	Yes
Eukaryota	Excavata	Metamonada	Metamonada_X	Parabasalia	-
Eukaryota	Excavata	Metamonada	Metamonada_X	Preaxostyla	Yes
Eukaryota	Haptista	Centroplasthelida	Centroplasthelida_X	Centroplasthelida_XX	Yes
Eukaryota	Haptista	Centroplasthelida	Centroplasthelida_X	Panacanthocystida	Yes
Eukaryota	Haptista	Centroplasthelida	Centroplasthelida_X	Pterocystida	Yes
Eukaryota	Haptista	Haptophyta	Haptophyta_X	Haptophyta_Clade_HAP1	-

Domain	Supergroup	Division	Subdivision	Class	Sequenced by PacBio
Eukaryota	Haptista	Haptophyta	Haptophyta_X	Haptophyta_Clade_HAP2	Yes
Eukaryota	Haptista	Haptophyta	Haptophyta_X	Haptophyta_Clade_HAP3	Yes
Eukaryota	Haptista	Haptophyta	Haptophyta_X	Haptophyta_Clade_HAP4	Yes
Eukaryota	Haptista	Haptophyta	Haptophyta_X	Haptophyta_Clade_HAP5	-
Eukaryota	Haptista	Haptophyta	Haptophyta_X	Haptophyta_XX	Yes
Eukaryota	Haptista	Haptophyta	Haptophyta_X	Pavlovophyceae	Yes
Eukaryota	Haptista	Haptophyta	Haptophyta_X	Prymnesiophyceae	Yes
Eukaryota	Obazoa	Apusomonada	Apusomonada_X	Apusomonadidae	Yes
Eukaryota	Obazoa	Breviatea	Breviatea_X	Breviatea_XX	Yes
Eukaryota	Obazoa	Breviatea	Breviatea_X	NAMAKO-1-lineage	-
Eukaryota	Obazoa	Breviatea	Breviatea_X	YS16Ec34-lineage	-
Eukaryota	Obazoa	Opisthokonta	Choanoflagellata	Choanoflagellata_X	Yes
Eukaryota	Obazoa	Opisthokonta	Filasterea	Filasterea_X	Yes
Eukaryota	Obazoa	Opisthokonta	Fungi	Ascomycota	Yes
Eukaryota	Obazoa	Opisthokonta	Fungi	Basidiomycota	Yes
Eukaryota	Obazoa	Opisthokonta	Fungi	Blastocladiomycota	Yes
Eukaryota	Obazoa	Opisthokonta	Fungi	Chytridiomycota	Yes
Eukaryota	Obazoa	Opisthokonta	Fungi	Fungi_X	Yes
Eukaryota	Obazoa	Opisthokonta	Fungi	Monoblepharidomycetes	Yes
Eukaryota	Obazoa	Opisthokonta	Fungi	Mucoromycota	Yes
Eukaryota	Obazoa	Opisthokonta	Fungi	Neocallimastigaceae	-
Eukaryota	Obazoa	Opisthokonta	Fungi	Olpidium_class	-
Eukaryota	Obazoa	Opisthokonta	Fungi	Opisthosporidia	Yes
Eukaryota	Obazoa	Opisthokonta	Fungi	Zoopagomycota	Yes
Eukaryota	Obazoa	Opisthokonta	Ichthyosporea	Dermocystida	Yes
Eukaryota	Obazoa	Opisthokonta	Ichthyosporea	Ichthyophonida	Yes
Eukaryota	Obazoa	Opisthokonta	Ichthyosporea	Ichthyosphonida	Yes
Eukaryota	Obazoa	Opisthokonta	Metazoa	Acanthocephala	Yes
Eukaryota	Obazoa	Opisthokonta	Metazoa	Annelida	Yes
Eukaryota	Obazoa	Opisthokonta	Metazoa	Arthropoda	Yes
Eukaryota	Obazoa	Opisthokonta	Metazoa	Brachiopoda	Yes
Eukaryota	Obazoa	Opisthokonta	Metazoa	Bryozoa	Yes
Eukaryota	Obazoa	Opisthokonta	Metazoa	Cephalochordata	-
Eukaryota	Obazoa	Opisthokonta	Metazoa	Chaetognatha	Yes

Domain	Supergroup	Division	Subdivision	Class	Sequenced by PacBio
Eukaryota	Obazoa	Opisthokonta	Metazoa	Cnidaria	Yes
Eukaryota	Obazoa	Opisthokonta	Metazoa	Craniata	-
Eukaryota	Obazoa	Opisthokonta	Metazoa	Ctenophora	Yes
Eukaryota	Obazoa	Opisthokonta	Metazoa	Cycliophora	-
Eukaryota	Obazoa	Opisthokonta	Metazoa	Echinodermata	Yes
Eukaryota	Obazoa	Opisthokonta	Metazoa	Entoprocta	-
Eukaryota	Obazoa	Opisthokonta	Metazoa	Gastrotricha	Yes
Eukaryota	Obazoa	Opisthokonta	Metazoa	Gnathostomulida	-
Eukaryota	Obazoa	Opisthokonta	Metazoa	Hemichordata	-
Eukaryota	Obazoa	Opisthokonta	Metazoa	Kinorhyncha	-
Eukaryota	Obazoa	Opisthokonta	Metazoa	Loricifera	-
Eukaryota	Obazoa	Opisthokonta	Metazoa	Mesozoa	-
Eukaryota	Obazoa	Opisthokonta	Metazoa	Metazoa_X	-
Eukaryota	Obazoa	Opisthokonta	Metazoa	Micrognathozoa	-
Eukaryota	Obazoa	Opisthokonta	Metazoa	Mollusca	Yes
Eukaryota	Obazoa	Opisthokonta	Metazoa	Myxozoa	Yes
Eukaryota	Obazoa	Opisthokonta	Metazoa	Myzostomida	-
Eukaryota	Obazoa	Opisthokonta	Metazoa	Nematoda	Yes
Eukaryota	Obazoa	Opisthokonta	Metazoa	Nematomorpha	Yes
Eukaryota	Obazoa	Opisthokonta	Metazoa	Nemertea	-
Eukaryota	Obazoa	Opisthokonta	Metazoa	Onychophora	-
Eukaryota	Obazoa	Opisthokonta	Metazoa	Placozoa	-
Eukaryota	Obazoa	Opisthokonta	Metazoa	Platyhelminthes	Yes
Eukaryota	Obazoa	Opisthokonta	Metazoa	Porifera	-
Eukaryota	Obazoa	Opisthokonta	Metazoa	Priapulida	-
Eukaryota	Obazoa	Opisthokonta	Metazoa	Rotifera	Yes
Eukaryota	Obazoa	Opisthokonta	Metazoa	Tardigrada	Yes
Eukaryota	Obazoa	Opisthokonta	Metazoa	Urochordata	Yes
Eukaryota	Obazoa	Opisthokonta	Metazoa	Xenoturbellida	-
Eukaryota	Obazoa	Opisthokonta	Opisthokonta_X	Opisthokonta_XX	Yes
Eukaryota	Obazoa	Opisthokonta	Pluriformea	Corallochytrea	-
Eukaryota	Obazoa	Opisthokonta	Pluriformea	Pluriformea_X	Yes
Eukaryota	Obazoa	Opisthokonta	Rotosphaerida	Fonticulea	-
Eukaryota	Obazoa	Opisthokonta	Rotosphaerida	Nucleariidea	Yes

Domain	Supergroup	Division	Subdivision	Class	Sequenced by PacBio
Eukaryota	TSAR	Alveolata	Alveolata_X	Alveolata_XX	-
Eukaryota	TSAR	Alveolata	Apicomplexa	Apicomplexa_X	Yes
Eukaryota	TSAR	Alveolata	Apicomplexa	Coccidiomorphea	Yes
Eukaryota	TSAR	Alveolata	Apicomplexa	Colpodellidea	Yes
Eukaryota	TSAR	Alveolata	Apicomplexa	Gregarinomorphea	Yes
Eukaryota	TSAR	Alveolata	Ciliophora	Armophorea	Yes
Eukaryota	TSAR	Alveolata	Ciliophora	Cariacotrichea	-
Eukaryota	TSAR	Alveolata	Ciliophora	Ciliophora_X	Yes
Eukaryota	TSAR	Alveolata	Ciliophora	Colpodea	Yes
Eukaryota	TSAR	Alveolata	Ciliophora	CONTH_1	-
Eukaryota	TSAR	Alveolata	Ciliophora	CONTH_2	Yes
Eukaryota	TSAR	Alveolata	Ciliophora	CONTH_3	-
Eukaryota	TSAR	Alveolata	Ciliophora	CONTH_4	Yes
Eukaryota	TSAR	Alveolata	Ciliophora	CONTH_5	Yes
Eukaryota	TSAR	Alveolata	Ciliophora	CONTH_6	-
Eukaryota	TSAR	Alveolata	Ciliophora	CONTH_7	Yes
Eukaryota	TSAR	Alveolata	Ciliophora	CONTH_8	-
Eukaryota	TSAR	Alveolata	Ciliophora	CONThreeP	Yes
Eukaryota	TSAR	Alveolata	Ciliophora	Cyclotrichium_like_organism	-
Eukaryota	TSAR	Alveolata	Ciliophora	Heterotrichea	Yes
Eukaryota	TSAR	Alveolata	Ciliophora	Karyorelictea	-
Eukaryota	TSAR	Alveolata	Ciliophora	Litostomatea	Yes
Eukaryota	TSAR	Alveolata	Ciliophora	Nassophorea	Yes
Eukaryota	TSAR	Alveolata	Ciliophora	Oligohymenophorea	Yes
Eukaryota	TSAR	Alveolata	Ciliophora	Phyllopharyngea	Yes
Eukaryota	TSAR	Alveolata	Ciliophora	Plagiopylea	Yes
Eukaryota	TSAR	Alveolata	Ciliophora	Prostomatea_1	Yes
Eukaryota	TSAR	Alveolata	Ciliophora	Prostomatea_2	Yes
Eukaryota	TSAR	Alveolata	Ciliophora	Prostomatea_3	Yes
Eukaryota	TSAR	Alveolata	Ciliophora	Spirotrichea	Yes
Eukaryota	TSAR	Alveolata	Colponemidia	Colponemidia_X	-
Eukaryota	TSAR	Alveolata	Dinoflagellata	Dinophyceae	Yes
Eukaryota	TSAR	Alveolata	Dinoflagellata	Dinophyta_X	Yes
Eukaryota	TSAR	Alveolata	Dinoflagellata	Noctilucophyceae	-

Domain	Supergroup	Division	Subdivision	Class	Sequenced by PacBio
Eukaryota	TSAR	Alveolata	Dinoflagellata	Oxyrrhea	-
Eukaryota	TSAR	Alveolata	Dinoflagellata	Syndiniales	Yes
Eukaryota	TSAR	Alveolata	Perkinsea	Perkinsea_X	-
Eukaryota	TSAR	Alveolata	Perkinsea	Perkinsida	Yes
Eukaryota	TSAR	Rhizaria	Cercozoa	Cercozoa_X	-
Eukaryota	TSAR	Rhizaria	Cercozoa	Chlorarachniophyceae	Yes
Eukaryota	TSAR	Rhizaria	Cercozoa	Endomyxa-Ascetosporea	Yes
Eukaryota	TSAR	Rhizaria	Cercozoa	Endomyxa	Yes
Eukaryota	TSAR	Rhizaria	Cercozoa	Endomyxa-Gromiidea	Yes
Eukaryota	TSAR	Rhizaria	Cercozoa	Endomyxa-Phytomyxea	Yes
Eukaryota	TSAR	Rhizaria	Cercozoa	Endomyxa	Yes
Eukaryota	TSAR	Rhizaria	Cercozoa	Filosa	Yes
Eukaryota	TSAR	Rhizaria	Cercozoa	Filosa-Granofilosea	Yes
Eukaryota	TSAR	Rhizaria	Cercozoa	Filosa-Imbricatea	Yes
Eukaryota	TSAR	Rhizaria	Cercozoa	Filosa-Metromonadea	-
Eukaryota	TSAR	Rhizaria	Cercozoa	Filosa-Sarcomonadea	Yes
Eukaryota	TSAR	Rhizaria	Cercozoa	Filosa-Thecofilosea	Yes
Eukaryota	TSAR	Rhizaria	Cercozoa	Novel-clade-10-12	Yes
Eukaryota	TSAR	Rhizaria	Cercozoa	Phaeodarea	Yes
Eukaryota	TSAR	Rhizaria	Foraminifera	Foraminifera_X	-
Eukaryota	TSAR	Rhizaria	Foraminifera	Globothalamea	Yes
Eukaryota	TSAR	Rhizaria	Foraminifera	Monothalamids	Yes
Eukaryota	TSAR	Rhizaria	Foraminifera	Tubothalamea	Yes
Eukaryota	TSAR	Rhizaria	Radiolaria	Acantharea	Yes
Eukaryota	TSAR	Rhizaria	Radiolaria	Polycystinea	Yes
Eukaryota	TSAR	Rhizaria	Radiolaria	RAD-A	Yes
Eukaryota	TSAR	Rhizaria	Radiolaria	RAD-B	Yes
Eukaryota	TSAR	Rhizaria	Radiolaria	RAD-C	Yes
Eukaryota	TSAR	Rhizaria	Radiolaria	Radiolaria_X	Yes
Eukaryota	TSAR	Stramenopiles	Bigyra	Bigyra_X	-
Eukaryota	TSAR	Stramenopiles	Bigyra	Opalozoa	Yes
Eukaryota	TSAR	Stramenopiles	Bigyra	Sagenista	Yes
Eukaryota	TSAR	Stramenopiles	Gyrista	Aurearenophyceae	-
Eukaryota	TSAR	Stramenopiles	Gyrista	Bolidophyceae	Yes

Domain	Supergroup	Division	Subdivision	Class	Sequenced by PacBio
Eukaryota	TSAR	Stramenopiles	Gyrista	Chrysista	Yes
Eukaryota	TSAR	Stramenopiles	Gyrista	Chrysomerophyceae	-
Eukaryota	TSAR	Stramenopiles	Gyrista	Chrysophyceae	Yes
Eukaryota	TSAR	Stramenopiles	Gyrista	Developea	-
Eukaryota	TSAR	Stramenopiles	Gyrista	Diatomeae	Yes
Eukaryota	TSAR	Stramenopiles	Gyrista	Dictyochophyceae	Yes
Eukaryota	TSAR	Stramenopiles	Gyrista	Eustigmatophyceae	Yes
Eukaryota	TSAR	Stramenopiles	Gyrista	Gyrista_X	Yes
Eukaryota	TSAR	Stramenopiles	Gyrista	Hyphochytriomyceta	Yes
Eukaryota	TSAR	Stramenopiles	Gyrista	MOCH-1	-
Eukaryota	TSAR	Stramenopiles	Gyrista	MOCH-2	Yes
Eukaryota	TSAR	Stramenopiles	Gyrista	MOCH-4	Yes
Eukaryota	TSAR	Stramenopiles	Gyrista	Pelagophyceae	Yes
Eukaryota	TSAR	Stramenopiles	Gyrista	Peronosporomycetes	Yes
Eukaryota	TSAR	Stramenopiles	Gyrista	Phaeophyceae	Yes
Eukaryota	TSAR	Stramenopiles	Gyrista	Phaeothamniophyceae	-
Eukaryota	TSAR	Stramenopiles	Gyrista	Picophagea	-
Eukaryota	TSAR	Stramenopiles	Gyrista	Pinguiophyceae	-
Eukaryota	TSAR	Stramenopiles	Gyrista	Pirsoniales	Yes
Eukaryota	TSAR	Stramenopiles	Gyrista	Raphidophyceae	Yes
Eukaryota	TSAR	Stramenopiles	Gyrista	Schizocladiophyceae	-
Eukaryota	TSAR	Stramenopiles	Gyrista	Synchromophyceae	-
Eukaryota	TSAR	Stramenopiles	Gyrista	Xanthophyceae	Yes
Eukaryota	TSAR	Stramenopiles	Stramenopiles_X	Stramenopiles-Group-2	-
Eukaryota	TSAR	Stramenopiles	Stramenopiles_X	Stramenopiles-Group-3	Yes
Eukaryota	TSAR	Stramenopiles	Stramenopiles_X	Stramenopiles-Group-4	-
Eukaryota	TSAR	Stramenopiles	Stramenopiles_X	Stramenopiles-Group-6	-
Eukaryota	TSAR	Stramenopiles	Stramenopiles_X	Stramenopiles-Group-7	-
Eukaryota	TSAR	Stramenopiles	Stramenopiles_X	Stramenopiles-Group-8	-
Eukaryota	TSAR	Stramenopiles	Stramenopiles_X	Stramenopiles-Group-9	Yes
Eukaryota	TSAR	Stramenopiles	Stramenopiles_X	Stramenopiles_XX	Yes
Eukaryota	TSAR	Telonemia	Telonemia_X	Telonemia_XX	Yes

Supplementary Table 3. Details of the short-read metabarcoding datasets used in this study. ID refers to the dataset ID in metaPR2[47]. Number of ASVs is obtained after processing the raw reads with DADA2[48].

No.	ID	Dataset name	Ref	Env level 1	Env level 2	Description	Location	No. of samples	Sampling date	Sequencing technology	Fwd primer	Rev primer	Primer set in PR² primer database[5]	Number of ASVs	Bioproject	Repo
1	1	Ocean Sampling Day	49	marine	euphotic	global coastal waters	global	155	2014-06-21	Illumina	TAReuk454FWD1	V4 18S Next.Rev	16	12451	PRJEB8682	ENA
2	2	Ocean Sampling Day	49	marine	euphotic	global coastal waters	global	138	2015-06-21	Illumina	TAReuk454FWD1	V4 18S Next.Rev	16	11083	PRJEB8682	ENA
3	3	Ocean Sampling Day	49	marine	euphotic	global coastal waters	global	29	2014-06-21	Illumina	TAReuk454FWD1	V4 18S Next.Rev	16	13405	PRJEB8682	ENA
4	34	Malaspina Expedition	45	marine	euphotic + aphotic	global marine waters	global	56 + 124	2010-2011	Illumina	TAReuk454FWD1	TAReukREV3	8	17656	PRJEB23771	ENA
5	35	Malaspina Expedition	50	marine	euphotic	global marine waters	global	119	2010-2011	Illumina	TAReuk454FWD1	TAReukREV3	8	15338	PRJEB23913	ENA
6	53	Biomarks	51	marine	euphotic	European coastal waters	coast of Europe	50	2010-2011	454	TAReuk454FWD1	TAReukREV3	8	9097	PRJEB9133	NCBI
7	69	Mariana Trench 1	17	marine	aphotic	deep sea trench	Mariana Trench	32	2016	Illumina	TAReuk454FWD1	TAReukREV3	8	5842	SRP141405	NCBI
8	70	Mariana Trench 2	52	marine	aphotic	deep sea trench	Mariana Trench	12	2016	Illumina	3NDF	V4_euk_R2	14	1115	PRJNA399026	NCBI
9	150	Saint-Charles River	53	non-marine	freshwater	river	Canada	144	2016-2017	Illumina	E572F	E1009R	17	5584	PRJEB36925	NCBI
10	183	Lake Fuxian	54	non-marine	freshwater	lake	China	21	2015	Illumina	NSF573	NSR951	25	3224	PRJNA534173	NCBI
11	185	Lake Chaohu	55	non-marine	freshwater	lake	China	25	2014-2015	Illumina	NSF573	NSR951	25	1733	PRJNA534176, PRJNA330896	NCBI
12	195	Lake Baikal	56	non-marine	freshwater	lake	Siberia	23	2013	Illumina	TAReukFWD1	TAReukREV3	154	1125	PRJEB24415	ENA
13	196	Chevreuse	57	non-marine	freshwater	ponds	France	12	2012	454	EK-565F	EUK1134-R	24	807	PRJNA259710	NCBI
14	197	Mountain Lakes	58	non-marine	freshwater	high-altitude lakes	Austria, Chile, Ethiopia	19	2013	Illumina	TAReuk454FWD1	TAReukREV3	8	25935	SRP065150	NCBI
15	198	Lake Garda	59	non-marine	freshwater	alpine lake	Italy	64	2014-2015	Illumina	TAReuk454FWD1	V4 18S Next.Rev	16	1173	PRJEB36925	ENA
16	199	Neotropical Soils	60	non-marine	soil	Neotropical forest soils	Central + South America	154	2012-2013	Illumina	TAReuk454FWD1	TAReukREV3	8	28474	PRJNA317860	NCBI
17	200	Parana River	61	non-marine	freshwater	floodplain lakes	South America	10	2012	Illumina	528F	Nex_18S_0964_R	22	7540	PRJEB23471; PRJEB23493	ENA
18	201	Swiss Soils	62	non-marine	soil	soil	Switzerland	165	2013	Illumina	TAReuk454FWD1	TAReukREV3	8	49669	PRJEB30010	ENA
19	203	Scandinavian Lakes	63	non-marine	freshwater	lakes	Scandinavia	87	2011	454	TAReuk454FWD1	TAReukREV3	8	2782	7s6s8	Dryad
20	204	Global Soils	64	non-marine	soil	global soils	global	40	-	454	515F	119r	18	663	-	-
21	205	Tara Oceans V4	65	marine	euphotic	global marine waters	global	67	2009-2013	Illumina	TAReuk454FWD1	TAReukREV3	8	20180	PRJEB6610	ENA
22	206	Tara Arctic V4	65	marine	euphotic	Arctic Ocean	Arctic	14	2009-2013	Illumina	TAReuk454FWD1	TAReukREV3	8	2209	PRJEB9737	ENA

Supplementary Table 4. Environmental diversity collected from public datasets. Reads were processed using DADA2[48]. OTUs were conservatively filtered to reduce the number of "contaminant" taxa being kept, and only OTUs that had at least 100 reads or were present in two or more distinct samples were retained.

Sample type	No. of samples	No. of processed reads	No. of ASVs	No. of OTUs (97% similarity)	No. of OTUs post filteration
**soil**	359	127,197,084	78,806	20,474	11,935
**freshwater**	405	11,985,510	49,903	10,067	3,788
**marine euphotic**	628	83,409,837	90,017	20,440	9,977
**marine aphotic**	168	11,500,621	14,205	4,268	2,518
**non-marine (all)**	764	139,182,594	128,709	30,541	15,723
**Marine (all)**	796	94,910,458	104,222	24,708	12,495

References

1. Lozupone, C. & Knight, R. UniFrac: A new phylogenetic method for comparing microbial communities. *Appl. Environ. Microbiol.* **71**, 8228–8235 (2005).
2. Sieber, G., Beisser, D., Bock, C. & Boenigk, J. Protistan and fungal diversity in soils and freshwater lakes are substantially different. *Sci. Rep.* **10**, (2020).
3. Bass, D. *et al.* Rhizarian 'Novel Clade 10' Revealed as Abundant and Diverse Planktonic and Terrestrial Flagellates, including Aquavolon n. gen. *J. Eukaryot. Microbiol.* **65**, 828–842 (2018).
4. Shen, W., Le, S., Li, Y. & Hu, F. SeqKit: A Cross-Platform and Ultrafast Toolkit for FASTA/Q File Manipulation. *PLoS One* **11**, e0163962 (2016).
5. Vaulot, D., Geisen, S., Mahé, F. & Bass, D. pr2-primers: An 18S rRNA primer database for protists. *Mol. Ecol. Resour.* **22**, 168–179 (2022).
6. Martin, M. Cutadapt removes adapter sequences from high-throughput sequencing reads. *EMBnet.journal* **17**, 10 (2011).
7. Smith, S. A. & O'Meara, B. C. TreePL: Divergence time estimation using penalized likelihood for large phylogenies. *Bioinformatics* **28**, 2689–2690 (2012).
8. Strassert, J. F. H., Irisarri, I., Williams, T. A. & Burki, F. A molecular timescale for eukaryote evolution with implications for the origin of red algal-derived plastids. *Nat. Commun.* **12**, 1–13 (2021).
9. Žerdoner Čalasan, A., Kretschmann, J. & Gottschling, M. They are young, and they are many: dating freshwater lineages in unicellular dinophytes. *Environ. Microbiol.* **21**, 4125–4135 (2019).
10. Batten, D. J. Cretaceous freshwater dinoflagellates. *Cretac. Res.* **10**, 271–273 (1989).
11. Gray, J. Evolution of the freshwater ecosystem: The fossil record. *Palaeogeogr. Palaeoclimatol. Palaeoecol.* **62**, 1–214 (1988).
12. Pattengale, N. D., Alipour, M., Bininda-Emonds, O. R. P., Moret, B. M. E. & Stamatakis, A. How many bootstrap replicates are necessary? *J. Comput. Biol.* **17**, 337–354 (2010).
13. Jones, E. B. G. *et al.* An online resource for marine fungi. *Fungal Divers. 2019 961* **96**, 347–433 (2019).
14. Richards, T. A., Jones, M. D. M., Leonard, G. & Bass, D. Marine Fungi: Their Ecology and Molecular Diversity. *Ann. Rev. Mar. Sci.* **4**, 495–522 (2012).
15. Amend, A. From Dandruff to Deep-Sea Vents: Malassezia-like Fungi Are Ecologically Hyper-diverse. *PLoS Pathog.* **10**, e1004277 (2014).
16. Orsi, W., Biddle, J. F. & Edgcomb, V. Deep Sequencing of Subseafloor Eukaryotic rRNA Reveals Active Fungi across Marine Subsurface Provinces. *PLoS One* **8**, e56335 (2013).
17. Jing, H., Zhang, Y., Li, Y., Zhu, W. & Liu, H. Spatial Variability of Picoeukaryotic Communities in the Mariana Trench. *Sci. Rep.* **8**, 15357 (2018).
18. Bik, H. M., Halanych, K. M., Sharma, J. & Thomas, W. K. Dramatic Shifts in Benthic Microbial Eukaryote Communities following the Deepwater Horizon Oil Spill. *PLoS One* **7**, e38550 (2012).
19. Tisthammer, K. H., Cobian, G. M. & Amend, A. S. Global biogeography of marine fungi is shaped by the environment. *Fungal Ecol.* **19**, 39–46 (2016).
20. Marchese, P. *et al.* Fungi populate deep-sea coral gardens as well as marine sediments in the Irish Atlantic Ocean. *Environ. Microbiol.* **23**, 4168–4184 (2021).
21. Bochdansky, A. B., Clouse, M. A. & Herndl, G. J. Eukaryotic microbes, principally fungi and labyrinthulomycetes, dominate biomass on bathypelagic marine snow. *ISME J.* **11**,

362–373 (2017).
22. Bass, D. *et al.* Yeast forms dominate fungal diversity in the deep oceans. *Proc. R. Soc. B Biol. Sci.* **274**, 3069 (2007).
23. Kilias, E. S. *et al.* Chytrid fungi distribution and co-occurrence with diatoms correlate with sea ice melt in the Arctic Ocean. *Commun. Biol. 2020 31* **3**, 1–13 (2020).
24. Hassett, B. T. & Gradinger, R. Chytrids dominate arctic marine fungal communities. *Environ. Microbiol.* **18**, 2001–2009 (2016).
25. Gutiérrez, M. H., Pantoja, S., Tejos, E. & Quiñones, R. A. The role of fungi in processing marine organic matter in the upwelling ecosystem off Chile. *Mar. Biol.* **158**, 205–219 (2011).
26. Cunliffe, M., Hollingsworth, A., Bain, C., Sharma, V. & Taylor, J. D. Algal polysaccharide utilisation by saprotrophic planktonic marine fungi. *Fungal Ecol.* **30**, 135–138 (2017).
27. Pachiadaki, M. G., Rédou, V., Beaudoin, D. J., Burgaud, G. & Edgcomb, V. P. Fungal and prokaryotic activities in the Marine subsurface biosphere at Peru Margin and canterbury basin inferred from RNA-based analyses and microscopy. *Front. Microbiol.* **7**, (2016).
28. Orsi, W. D., Edgcomb, V. P., Christman, G. D. & Biddle, J. F. Gene expression in the deep biosphere. *Nat. 2013 4997457* **499**, 205–208 (2013).
29. Karsten, H. XXXIII.—Contribution towards the knowledge of the Rhynchoprion penetrans. *Ann. Mag. Nat. Hist.* **15**, 293–312 (1865).
30. Klaveness, D. Collodictyon triciliatum H.J. Carter (1865) - a common but fixation-sensitive algivorous flagellate from the limnopelagial. *Nord. J. Freshw. Res.* **70**, 3–11 (1995).
31. Zhao, S. *et al.* Collodictyon--an ancient lineage in the tree of eukaryotes. *Mol. Biol. Evol.* **29**, 1557–1568 (2012).
32. Orr, R. J. S. *et al.* Enigmatic Diphyllatea eukaryotes: culturing and targeted PacBio RS amplicon sequencing reveals a higher order taxonomic diversity and global distribution. *BMC Evol. Biol.* **18**, 115 (2018).
33. Glücksman, E. *et al.* The Novel Marine Gliding Zooflagellate Genus Mantamonas (Mantamonadida ord. n.: Apusozoa). *Protist* **162**, 207–221 (2011).
34. Kim, E., Sprung, B., Duhamel, S., Filardi, C. & Shin, M. K. Oligotrophic lagoons of the South Pacific Ocean are home to a surprising number of novel eukaryotic microorganisms. *Environ. Microbiol.* **18**, 4549–4563 (2016).
35. Hehenberger, E. *et al.* Novel Predators Reshape Holozoan Phylogeny and Reveal the Presence of a Two-Component Signaling System in the Ancestor of Animals. *Curr. Biol.* **27**, 2043-2050.e6 (2017).
36. Janouškovec, J. *et al.* A New Lineage of Eukaryotes Illuminates Early Mitochondrial Genome Reduction. *Curr. Biol.* **27**, 3717-3724.e5 (2017).
37. Kies, L. & Kremer, B. P. Typification of the Glaucocystophyta. *Taxon* **35**, 128–133 (1986).
38. Dittami, S. M., Heesch, S., Olsen, J. L. & Collén, J. Transitions between marine and freshwater environments provide new clues about the origins of multicellular plants and algae. *J. Phycol.* **53**, 731–745 (2017).
39. Sánchez-Baracaldo, P., Raven, J. A., Pisani, D. & Knoll, A. H. Early photosynthetic eukaryotes inhabited low-salinity habitats. *Proc. Natl. Acad. Sci.* **114**, E7737–E7745 (2017).
40. Gawryluk, R. M. R. R. *et al.* Non-photosynthetic predators are sister to red algae.

Nature **572**, (2019).
41. Jamy, M. *et al.* Long-read metabarcoding of the eukaryotic rDNA operon to phylogenetically and taxonomically resolve environmental diversity. *Mol. Ecol. Resour.* **20**, 429–443 (2020).
42. Peura, S. *et al.* Ontogenic succession of thermokarst thaw ponds is linked to dissolved organic matter quality and microbial degradation potential. *Limnol. Oceanogr.* **65**, S248–S263 (2020).
43. Urbina, H., Scofield, D. G., Cafaro, M. & Rosling, A. DNA-metabarcoding uncovers the diversity of soil-inhabiting fungi in the tropical island of Puerto Rico. *Mycoscience* **57**, 217–227 (2016).
44. Kalsoom Khan, F. *et al.* Naming the untouchable – environmental sequences and niche partitioning as taxonomical evidence in fungi. *IMA Fungus* **11**, 1–12 (2020).
45. Giner, C. R. *et al.* Marked changes in diversity and relative activity of picoeukaryotes with depth in the world ocean. *ISME J.* **14**, 437–449 (2020).
46. Jamy, M. *et al.* Transitions datasets. *Figshare* https://doi.org/10.6084/m9.figshare.15164772.v1 (2021).
47. Vaulot, D. *et al.* metaPR2: a database of eukaryotic 18S rRNA metabarcodes with an emphasis on protists. *bioRxiv* 2022.02.04.479133 (2022).
48. Callahan, B. J. *et al.* DADA2: High-resolution sample inference from Illumina amplicon data. *Nat. Methods* **13**, 581–583 (2016).
49. Kopf, A. *et al.* The ocean sampling day consortium. *GigaScience* vol. 4 27 (2015).
50. Logares, R. *et al.* Disentangling the mechanisms shaping the surface ocean microbiota. *Microbiome 2020 81* **8**, 1–17 (2020).
51. Pernice, M. C., Logares, R., Guillou, L. & Massana, R. General Patterns of Diversity in Major Marine Microeukaryote Lineages. *PLoS One* **8**, e57170 (2013).
52. Xu, Z. *et al.* Vertical Distribution of Microbial Eukaryotes From Surface to the Hadal Zone of the Mariana Trench. *Front. Microbiol.* **9**, 2023 (2018).
53. Cruaud, P. *et al.* Annual Protist Community Dynamics in a Freshwater Ecosystem Undergoing Contrasted Climatic Conditions: The Saint-Charles River (Canada). *Front. Microbiol.* **10**, 2359 (2019).
54. Shi, X., Li, S., Li, H., Chen, F. & Wu, Q. The Community Structure of Picophytoplankton in Lake Fuxian, a Deep and Oligotrophic Mountain Lake. *Front. Microbiol.* **0**, 2016 (2019).
55. Shi, X., Li, S., Zhang, M., Liu, C. & Wu, Q. Temperature mainly determines the temporal succession of the photosynthetic picoeukaryote community in Lake Chaohu, a highly eutrophic shallow lake. *Sci. Total Environ.* **702**, 134803 (2020).
56. Annenkova, N. V., Giner, C. R. & Logares, R. Tracing the Origin of Planktonic Protists in an Ancient Lake. *Microorganisms* **8**, 543 (2020).
57. Simon, M. *et al.* Marked seasonality and high spatial variability of protist communities in shallow freshwater systems. *ISME J. 2015 99* **9**, 1941–1953 (2015).
58. Filker, S., Sommaruga, R., Vila, I. & Stoeck, T. Microbial eukaryote plankton communities of high-mountain lakes from three continents exhibit strong biogeographic patterns. *Mol. Ecol.* **25**, 2286–2301 (2016).
59. Salmaso, N., Boscaini, A. & Pindo, M. Unraveling the Diversity of Eukaryotic Microplankton in a Large and Deep Perialpine Lake Using a High Throughput Sequencing Approach. *Front. Microbiol.* **0**, 789 (2020).
60. Mahé, F. *et al.* Parasites dominate hyperdiverse soil protist communities in

Neotropical rainforests. *Nat. Ecol. Evol.* **1**, 0091 (2017).
61. Arroyo, A. S., López-Escardó, D., Kim, E., Ruiz-Trillo, I. & Najle, S. R. Novel Diversity of Deeply Branching Holomycota and Unicellular Holozoans Revealed by Metabarcoding in Middle Paraná River, Argentina. *Front. Ecol. Evol.* **0**, 99 (2018).
62. Seppey, C. V. W. *et al.* Soil protist diversity in the Swiss western Alps is better predicted by topo-climatic than by edaphic variables. *J. Biogeogr.* **47**, 866–878 (2020).
63. Khomich, M., Kauserud, H., Logares, R., Rasconi, S. & Andersen, T. Planktonic protistan communities in lakes along a large-scale environmental gradient. *FEMS Microbiol. Ecol.* **93**, 231 (2017).
64. Bates, S. T. *et al.* Global biogeography of highly diverse protistan communities in soil. *ISME J.* **7**, 652–659 (2013).
65. Ibarbalz, F. M. *et al.* Global Trends in Marine Plankton Diversity across Kingdoms of Life. *Cell* **179**, 1084-1097.e21 (2019).
