# Supplementary material for: Global patterns and rates of habitat transitions across the eukaryotic tree of life
Source: Nat Ecol Evol. 2022 Aug 4;6(10):1458–70. doi: 10.1038/s41559-022-01838-4 (PMC9525238; doi:10.1038/s41559-022-01838-4)
Supplement: Supplementary file 2 — Reporting Summary [file 41559_2022_1838_MOESM2_ESM.pdf]

nature portfolio

Corresponding author(s): Fabien Burki

Last updated by author(s): June 17, 2022

Reporting Summary

Nature Portfolio wishes to improve the reproducibility of the work that we publish. This form provides structure for consistency and transparency in reporting. For further information on Nature Portfolio policies, see our Editorial Policies and the Editorial Policy Checklist.

Statistics

For all statistical analyses, confirm that the following items are present in the figure legend, table legend, main text, or Methods section.

n/a	Confirmed	
☐	☒	The exact sample size (*n*) for each experimental group/condition, given as a discrete number and unit of measurement
☐	☒	A statement on whether measurements were taken from distinct samples or whether the same sample was measured repeatedly
☐	☒	The statistical test(s) used AND whether they are one- or two-sided<br>*Only common tests should be described solely by name; describe more complex techniques in the Methods section.*
☒	☐	A description of all covariates tested
☐	☒	A description of any assumptions or corrections, such as tests of normality and adjustment for multiple comparisons
☐	☒	A full description of the statistical parameters including central tendency (e.g. means) or other basic estimates (e.g. regression coefficient) AND variation (e.g. standard deviation) or associated estimates of uncertainty (e.g. confidence intervals)
☐	☒	For null hypothesis testing, the test statistic (e.g. *F*, *t*, *r*) with confidence intervals, effect sizes, degrees of freedom and *P* value noted<br>*Give P values as exact values whenever suitable.*
☐	☒	For Bayesian analysis, information on the choice of priors and Markov chain Monte Carlo settings
☒	☐	For hierarchical and complex designs, identification of the appropriate level for tests and full reporting of outcomes
☒	☐	Estimates of effect sizes (e.g. Cohen's *d*, Pearson's *r*), indicating how they were calculated

Our web collection on statistics for biologists contains articles on many of the points above.

Software and code

Policy information about availability of computer code

Data collection	No software was used for data collection.
Data analysis	SMRT Link v8.0.0.79519, mothur v1.39.5, DADA2 v1.14.1, VSEARCH v2.3.4, Blast v2.9.0, Barrnap v0.9, RAxML v8.2.10, RAxML-NG v0.9.0, EPA-ng v0.3.5, gappa v0.6.0, MAFFT v7.310, trimAl 1.2rev59, TreeShrink v1.3.5, cutadapt v3.3, PaPaRa v2.5, BayesTraits v3.0.2, pastml 1.9.30, PATHd8, Cytoscape v3.8.2. All custom scripts used in this study are available at https://doi.org/10.5281/zenodo.6656264 under a MIT license.

For manuscripts utilizing custom algorithms or software that are central to the research but not yet described in published literature, software must be made available to editors and reviewers. We strongly encourage code deposition in a community repository (e.g. GitHub). See the Nature Portfolio guidelines for submitting code & software for further information.

Data

Policy information about availability of data

All manuscripts must include a data availability statement. This statement should provide the following information, where applicable:

- Accession codes, unique identifiers, or web links for publicly available datasets
- A description of any restrictions on data availability
- For clinical datasets or third party data, please ensure that the statement adheres to our policy

New sequence data generated for this study were deposited at ENA under the accession number PRJEB45931, while data from Sequel I (generated in reference 28) were deposited under the accession number PRJEB25197. The PR2-transitions database, annotated 18S and 28S OTU sequences, clustered short read metabarcoding sequences used in this study, and all trees have been deposited in a Figshare repository (https://doi.org/10.6084/m9.figshare.15164772.v3). All custom code is available in a Zenodo repository (https://doi.org/10.5281/zenodo.6656264).

Field-specific reporting

Please select the one below that is the best fit for your research. If you are not sure, read the appropriate sections before making your selection.

☐ Life sciences ☐ Behavioural & social sciences ☒ Ecological, evolutionary & environmental sciences

For a reference copy of the document with all sections, see nature.com/documents/nr-reporting-summary-flat.pdf

Ecological, evolutionary & environmental sciences study design

All studies must disclose on these points even when the disclosure is negative.

Study description	A phylogenetic analysis to investigate habitat preference evolution across the tree of eukaryotes using long-read and short-read environmental sequencing data.
Research sample	Long-read metabarcoding data were generated from 21 samples covering soils, freshwater, marine euphotic, and marine aphotic habitats (details provided in Supplementary Table 1). With these data, we aimed to get a representative view of microbial eukaryotic diversity in each habitat. Existing short-read metabarcoding data were obtained from 22 publicly available datasets (details provided in Supplementary Table 3).
Sampling strategy	We ensured that the 21 samples sequenced in this study were not skewed towards a particular habitat, and used general eukaryotic primers were amplify the ribosomal DNA operon in order to capture the broad eukaryotic community. We then increased the covered eukaryotic diversity by using data from 22 publicly available short-read metabarcoding datasets spanning marine and non-marine habitats.
Data collection	Data were generated as part of this study, or downloaded from public databases.
Timing and spatial scale	Five freshwater samples (four planktonic and one sediment sample) were obtained from lakes in Sweden and permafrost thaw ponds in Canada in 2019 and 2014 respectively. Soil samples were sourced from Sweden (2019 and 2013), Puerto Rico (2013), UK (2015 and 2008), and Tibet (2011). Marine samples were obtained from the Mariana Trench (2016), the West Coast of Sweden (2019) and two stations from the Malaspina expedition (several depths; 2011). Sample coordinates and sampling dates are provided in Supplementary Table 1. It should be noted that sampling procedures for samples collected outside Sweden have been described in previous publications (see Supplementary Table 1 for references).
Data exclusions	We excluded 18S-28S sequences likely representing chimeras, artefacts from all samples and potential contaminants due to barcode mixing in one marine sample (Ms2-DCM), by manually inspecting 18S and 18S-28S phylogenies. To be conservative in what we considered to be "present" in a habitat, we excluded short-read OTUs that had low abundance (<100) or were only present in one environmental sample.
Reproducibility	All analyses are described in the Methods section with software versions and parameters used. Curated, labeled, 18S-28S OTU sequences are provided on FigShare, as are clustered short-read OTU sequences.
Randomization	We performed 1000 randomizations in the UniFrac analyses to test whether microbial communities from each habitat were phylogenetically distinct.
Blinding	No blinding was applied as no statistical tests were performed where blinding could be applied.

Did the study involve field work? ☒ Yes ☐ No

Field work, collection and transport

Field conditions	Marine surface waters, deep marine waters, soils, and freshwater lakes. Sampling dates and brief description of each sample is provided in Supplementary Table 1.
Location	Coordinates provided in Supplementary Table 1.
Access & import/export	Samples collected from outside Sweden have previously been described in previous publications (see Supplementary Table 1 for references); therefore no collecting permit was required for this study.
Disturbance	No disturbance was caused; only a small amount of water or soils were collected.

Reporting for specific materials, systems and methods

We require information from authors about some types of materials, experimental systems and methods used in many studies. Here, indicate whether each material, system or method listed is relevant to your study. If you are not sure if a list item applies to your research, read the appropriate section before selecting a response.

Materials & experimental systems

n/a	Involved in the study
☒	☐ Antibodies
☒	☐ Eukaryotic cell lines
☒	☐ Palaeontology and archaeology
☒	☐ Animals and other organisms
☒	☐ Human research participants
☒	☐ Clinical data
☒	☐ Dual use research of concern

Methods

n/a	Involved in the study
☒	☐ ChIP-seq
☒	☐ Flow cytometry
☒	☐ MRI-based neuroimaging
